# Molecular Insights Into β‐Glucuronidase Inhibition by *Alhagi Graecorum* Flavonoids: A Computational and Experimental Approach

**DOI:** 10.1002/open.202400325

**Published:** 2024-11-19

**Authors:** Emadeldin M. Kamel, Saleh Maodaa, Esam M. Al‐Shaebi, Al Mokhtar Lamsabhi

**Affiliations:** ^1^ Chemistry Department Faculty of Science Beni-Suef University Beni-Suef 62514 Egypt; ^2^ Department of Zoology College of Science King Saud University PO Box-2455 Riyadh 11451 Saudi Arabia; ^3^ Departamento de Química, Módulo 13 Universidad Autónoma de Madrid Campus de Excelencia UAM-CSIC Cantoblanco 28049 Madrid Spain; ^4^ Institute for Advanced Research in Chemical Sciences (IAdChem) Universidad Autónoma de Madrid 28049 Madrid Spain

**Keywords:** β-glucuronidase, Molecular dynamics simulations, *Alhagi graecorum*, *In vitro* study, Enzyme kinetics

## Abstract

In this study, we aimed to investigate the inhibitory mechanisms of β‐glucuronidase by flavonoids derived from *Alhagi graecorum* through both experimental and computational approaches. The activity of β‐glucuronidase was assessed using an in vitro enzyme inhibition assay, where myricetin and chrysoeriol were identified as potent inhibitors based on their low IC_50_ values. Kinetic studies were conducted to determine the inhibition type, revealing that both compounds exhibit noncompetitive inhibition of β‐glucuronidase‐catalyzed hydrolysis of PNPG. Molecular docking was employed to explore the binding affinities of the flavonoids, showing that myricetin formed the highest number of polar interactions with the enzyme. Additionally, molecular dynamics (MD) simulations were performed to evaluate the stability of the enzyme‐inhibitor complexes, demonstrating consistent trajectory behavior for both compounds, with significant energy stabilization. Interaction energy analyses highlighted the dominant role of electrostatic forces in myricetin′s inhibition mechanism, while Van der Waals forces were more prominent for chrysoeriol. The MM/PBSA method was used to calculate the binding free energies, with myricetin and chrysoeriol exhibiting the lowest values. Potential energy landscape analysis further revealed that β‐glucuronidase adopts a more closed conformation when bound to these inhibitors, limiting substrate access. These findings suggest that flavonoids from *Alhagi graecorum* hold promise for clinical applications, particularly in managing drug‐induced enteropathy.

## Graphical abstract

Five flavonoids were isolated from *Alhagi graecorum*, with myricetin and chrysoeriol exhibiting the lowest IC_50_ values against β‐glucuronidase. The study reveals that these flavonoids inhibit the enzyme activity through a noncompetitive inhibition mechanism. *In silico* analyses align with *in vitro* assay results, confirming the compounds’ inhibitory potential, suggesting their relevance for future therapeutic applications.

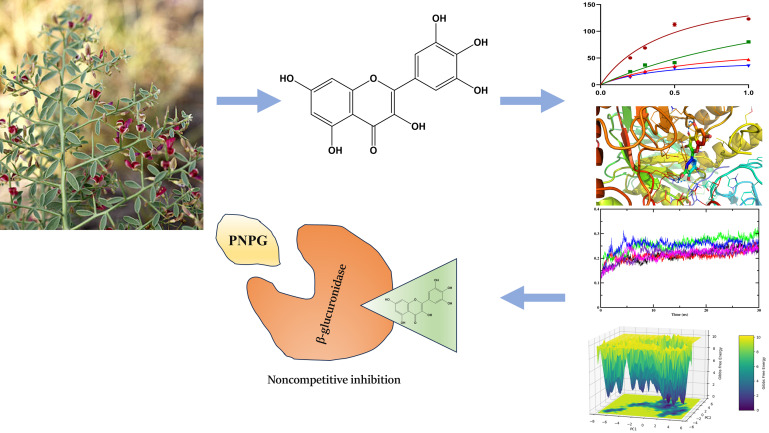

## 1 Introduction

Gut microbial β‐glucuronidase is a pivotal enzyme involved in the catalyzed biotransformation of many dietary and host‐derived glucuronides in the gastrointestinal tract.^[1]^ Located within the domain of phase I metabolism, β‐glucuronidase plays an important role as an essential member of the lysosomal glycosidase group.^[2]^ This enzyme is expressed within the gut microbiota and plays an important role in maintaining host health.^[3]^ The bioavailability and pharmacokinetic properties of many drugs, including xenobiotics and dietary natural products, are strongly affected by the catalytic activity of β‐glucuronidase.^[4]^ Also, this enzyme is responsible for metabolizing drugs, nutritional components, and essential endogenous compounds, thereby contributing significantly to overall physiological function.^[3]^ Dysregulation of β‐glucuronidase can lead to increased exposure to harmful substances, contributing to the development of cancer and other diseases.^[5]^ Thus, these carcinogenic compounds result in the pathogenesis of colorectal cancer and inflammatory bowel.^[5]^ Because of its crucial significance in gut diseases and health, the inhibition of gut bacterial β‐glucuronidase using natural products represents a potential strategy to regulate β‐glucuronidase function and alleviate correlated conditions.[^1^, ^6^] Furthermore, elucidating the inhibitory effects of flavonoids on β‐glucuronidase function may provide valuable information on innovative strategies for addressing gastrointestinal diseases and minimizing the likelihood of related illnesses.^[7]^

*Alhagi graecorum* (*A. graecorum*), commonly known as al‐akool, mannatree, or manna tree, is a perennial shrub from the Fabaceae family, widely distributed in arid and semi‐arid regions.^[8]^ This renowned medicinal plant is native to Saudi Arabia and extensively found across the Nile region, the Mediterranean Basin, eastern and western deserts, the Red Sea coast, and the Sinai Peninsula.^[8]^ This supple herb is characterized by its wide range of phytochemical constituents, including flavonoids, resins, alkaloids, saponins, triterpenes, and phenolic compounds.^[9]^ The bioactive secondary metabolites in this species significantly contribute to its pharmacological applications, including antioxidant, antimicrobial, anti‐inflammatory, and hepatoprotective activities.^[10]^ Dried specimens of *A. graecorum* are employed in traditional medicine as both a laxative and vermifuge for the treatment of bilharziasis and rheumatic pains.^[10]^ Additionally, *A. graecorum* finds application in addressing a spectrum of health issues including rheumatic pains, liver disorders, urinary tract infections, and assorted gastrointestinal discomforts.^[11]^ Extensive utilization of various plant parts is documented in the management of hemorrhoidal conditions across multiple studies.^[11]^ The rich diversity of phytochemicals in *A. graecorum*, coupled with its potent pharmacological activities, underscores its potential as a valuable medicinal resource.

Recently, the combination of computational techniques, particularly docking and molecular dynamics (MD) simulations, has provided deeper insights into figuring out the interaction mechanisms of drugs with biological macromolecules.^[13]^ Molecular docking provides valuable information about the binding profile of isolated flavonoids with the active site of the target enzyme, offering a rational basis for exploring the inhibitory activity of tested drugs.^[19]^ On the other hand, MD simulations allow for studying the dynamic interactions among proposed drugs and the target enzyme over time, providing a road map for assessing the energy variations and conformational variations of the flavonoids‐β‐glucuronidase complex.^[23]^ The aim of this study is to unravel the inhibitory potential of *A. graecorum* flavonoids against β‐glucuronidase employing a combination of *in vitro* assays, and *in silico* investigation. Through this thorough approach, we intend to figure out the conceivable interaction mechanisms of *A. graecorum* flavonoids and β‐glucuronidase, targeting the identification of potential lead drugs for the designing of novel therapeutic agents with the specific purpose of targeting β‐glucuronidase.

## Materials and Methods

### Phytochemical Studies

#### General Experimental Procedure

The NMR spectra of *A. graecorum* flavonoids (^1^H and ^13^C NMR) were recorded on the Bruker‐AVANCE III‐600 spectrometer. The chemical shifts were represented in δ (ppm), meanwhile, the coupling constants are represented as J in Hz. The UV‐Vis spectral data were calculated using Shimadzu UV 2201 spectrophotometer. The Rudolph Autopol III polarimeter was assigned as the polarimeter calculating the optical rotations. HREIMS and EIMS analyses were carried out utilizing an Agilent 6210 TOF instrument. Shimadzu FTIR‐8400 spectrometer was used for calculating the FTIR data on KBr pellets.

#### Plant Material, Extraction, and Isolation of Flavonoids

*A. graecorum* plant material was obtained for Beni‐Suef City in April 2023. Taxonomists from Beni‐Suef University conducted the plant identification. A voucher specimen of *A. graecorum* has been kept at our natural product lab under the authentication number BSU2023‐318. Next, the collected aerial parts were dried and grounded. Subsequently, an amount of 3.5 kg was subjected to extraction using 70% ethanol by cold maceration. Subsequently, the solvent was evaporated *in vacuo*, affording 234 g of crude extract. The obtained crude extract was partitioned between ethyl acetate, *n*‐butanol, and petroleum ether. The TLC profile of the ethyl acetate fraction displayed major spots. Then, 32 g of this fraction underwent chromatographic investigation over a silica gel column (110×3 cm, 0.80 kg) using the solvent system dichloromethane (DCM): ethanol (10 : 0 to 0 : 10) of increasing polarity as an eluent. To streamline the fractionation process, ultraviolet (UV) light was used to monitor the movement of compounds down the column. This allowed for the collection of nine subfractions, which were then combined into four distinct fractions (X1‐X4) based on their TLC profiles. Next, subfraction X1 was further purified utilizing a silica gel column (60×1.5 cm). The eluent was the system hexane/ethyl acetate with gradually increasing polarity. This process yielded eleven subfractions (Y1‐Y11). Since subfractions Y5‐Y9 exhibited similar TLC profiles, consequently, these fractions were combined and subjected to another round of chromatography on a Sephadex LH‐20 column eluted with methanol. This final purification step resulted in isolating the target compounds, **2** (21 mg) and **3** (19 mg). Subsequently, Y10 underwent chromatographic investigation on a Sephadex LH‐20 column and was eluted with a gradient of MeOH/H_2_O in an 80 : 20 ratio, affording the fractions Y10.1–Y10.5. Subfraction Y10.4 was subjected to additional purification through reversed‐phase preparative HPLC using a solvent system of acetonitrile/H_2_O (40 : 60), resulting in isolating compound **1** (23 mg). Following purification on a Sephadex LH‐20 column with methanol eluent, further chromatographic separation of fractions from Y10.5 yielded compound **5** (25 mg). Subfraction Y11 was then chromatographed on a Polyamide 6s column using methanol, resulting in four main subfractions (T1‐T4). Finally, semi‐preparative HPLC with 50% acetonitrile isolated compound **4** (26 mg) from subfraction T4.

### Inhibitory Activity Assay

#### Chemicals and Reagents

The substrate *p*‐Nitrophenyl‐*β*‐D‐glucuronide (PNPG) and the employed reference drug (−)‐Epigallocatechin gallate (EGCG) were purchased from Sigma‐Aldrich. For this study, β‐glucuronidase, the enzyme of interest, was purchased from Sigma (USA). All other chemicals and solvents used were in the highest purity state or HPLC grade. Five isolated flavonoids from *A. graecorum* were assigned for their β‐glucuronidase inhibitory efficacy. We prepared the β‐glucuronidase enzyme according to the method reported by Weng et al..^[27]^ A 100 mM stock solution of the substrate (PNPG) was prepared using HPLC‐grade DMSO and stored at −20 °C for subsequent utilization. Milli‐Q water was used exclusively for both the PNPG solution and the 0.1 M phosphate buffer solution (pH 7.4).

#### Inhibition Assay

To estimate the activities of tested flavonoids as inhibitors against β‐glucuronidase, we employed PNPG as the substrate. The assessment procedure followed well‐documented previously reported protocols.^[28]^ In brief, 96‐well plate assays were performed (100 μL/well) to assess β‐glucuronidase inhibition by flavonoids. Each well contained: β‐glucuronidase (2 μg/mL, 10 μL), flavonoid/EGCG inhibitor (10 μM, 10 μL), PNPG substrate (250 μM, 10 μL), and PBS buffer (pH 7.4, 70 μL). Reactions were done in triplicate. Enzyme activity was estimated by tracking PNP formation (OD₄₁₀ nm) after incubation (37 °C, 30 min). A standard curve (0–120 μM PNP) was used to quantify PNP. Relative activity (%) was calculated compared to the blank control. In addition, EGCG was employed as a reference drug. IC_50_ assays for flavonoid inhibition of β‐glucuronidase were conducted at at temperature of 37 °C for 30 minutes. Each reaction contained: β‐glucuronidase (2.0 μg/mL, 10 μL), flavonoid/EGCG (varying concentrations, 0.001–1000 μM, 10 μL), and PBS buffer (pH 7.4, 70 μL). Relative activity (%) was measured as enzyme activity with an inhibitor compared to activity without an inhibitor. A concentration‐response curve was constructed using relative activity data to determine IC_50_ values.

#### Inhibition Kinetics Studies

For myricetin and chrysoeriol (IC_50_<5 μM), we explored their inhibitory kinetics against β‐glucuronidase and determined inhibition constant (K_i_) values. We assessed the rate of hydrolysis of PNPG at various graients (200, 300, 500, and 1000 μM) with varying concentrations of myricetin, chrysoeriol, and EGCG. To analyze the binding interactions between the tested inhibitors and the target enzyme, we constructed Michaelis‐Menten and Lineweaver‐Burk plots. These plots helped us understand how the inhibitors affect the enzyme′s kinetics. The Lineweaver‐Burk plot′s intersection point was used to identify the inhibition mode (noncompetitive, competitive, uncompetitive, or mixed). Established methods were employed to determine the specific inhibition mode and K_i_ values.[^27^, ^28^]

#### Statistical Analysis

To ensure reproducibility, all data are presented as the mean value±standard deviation (SD). This reflects the average value obtained from three independent trials. IC_50_ values, signifying the inhibitor concentration required for half‐maximal response, were determined from the concentration‐response curves. The IC_50_ and inhibition constant (K_i_) values were estimated using nonlinear regression by the GraphPad Prism 9.0 software.

### Molecular Docking Analysis

Initially, the geometrical structures of studied flavonoids were optimized at the B3LYP level of theory using the 6–311G (d, p) basis set.^[29]^ These DFT calculations were performed using the Gaussian 16 software.^[32]^ We additionally performed vibrational frequency calculations using the same theoretical framework to ensure the optimized structures corresponded to true energetic minima (ground states) by verifying the nonexistence of imaginary frequencies. Docking calculations were performed using the AutoDock Vina package.^[33]^ The 3D crystal structure of β‐glucuronidase was downloaded from the Protein Data Bank (PDB ID: 3K4D). Autodock Tools (ADT) v1.5.6 program was used to prepare the enzyme and different ligands for the docking runs. The preparation process includes setting the grid box to the position of the native ligand, removal of nonstandard residues, polar hydrogen addition, and generation of different pdbqt files. To create high‐quality images and visualize the interactions between molecules at the binding site, we employed PyMOL version 2.4.

### Molecular Dynamics Simulations

Next, we selected the A. graecorum flavonoid‐β‐glucuronidase complexes (PDB ID: 3K4D). These complexes had the highest binding affinities predicted by molecular docking for further MD simulations. CGenFF (https://cgenff.com/) was employed to build the topology and geometric parameters for the various flavonoid ligands. The obtained parameters were then incorporated into the existing topology of the enzyme. The GROMACS 2022.4 software was employed to perform 30 ns MD.^[35]^ The simulations utilized the CHARMM36m force field.^[37]^ Both the free enzyme and different flavonoid complexes were solvated in a dodecahedron box with periodic boundary conditions, affording a volume of approximately 842.77 nm^3^ of the box. The water model used was the CHARMM‐modified TIP3P, and electrical neutrality was maintained by adding 24 sodium counterions.^[38]^ To eliminate unfavorable interactions, a 10 ps energy minimization with the steepest descent was performed.^[39]^ This was followed by a two‐step equilibration process at 300 K for 100 ps each in the NVT and NPT ensembles.^[40]^ Eventually, the production MD simulations were run for 30 ns at a pressure of 1 bar and a temperature of 300 K.

We used the Molecular Mechanics/Poisson‐Boltzmann Surface Area (MM/PBSA) method to estimate the binding free energies between the tested inhibitors and the target enzyme. This method combines molecular mechanics energies with solvation‐free energies calculated using the Poisson‐Boltzmann equation and a surface area term accounting for non‐polar interactions. MD simulation trajectories were used as input, with snapshots extracted at regular intervals. These snapshots were then analyzed using MM/PBSA, providing an average binding free energy across the simulation. The calculations were performed with the gmx_MMPBSA tool, ensuring a robust and reliable estimation of the ligand‐enzyme binding free energies.^[41]^

## 2 Results and Discussion

### 2.1 Phytochemical Analysis

**Figure 1:**
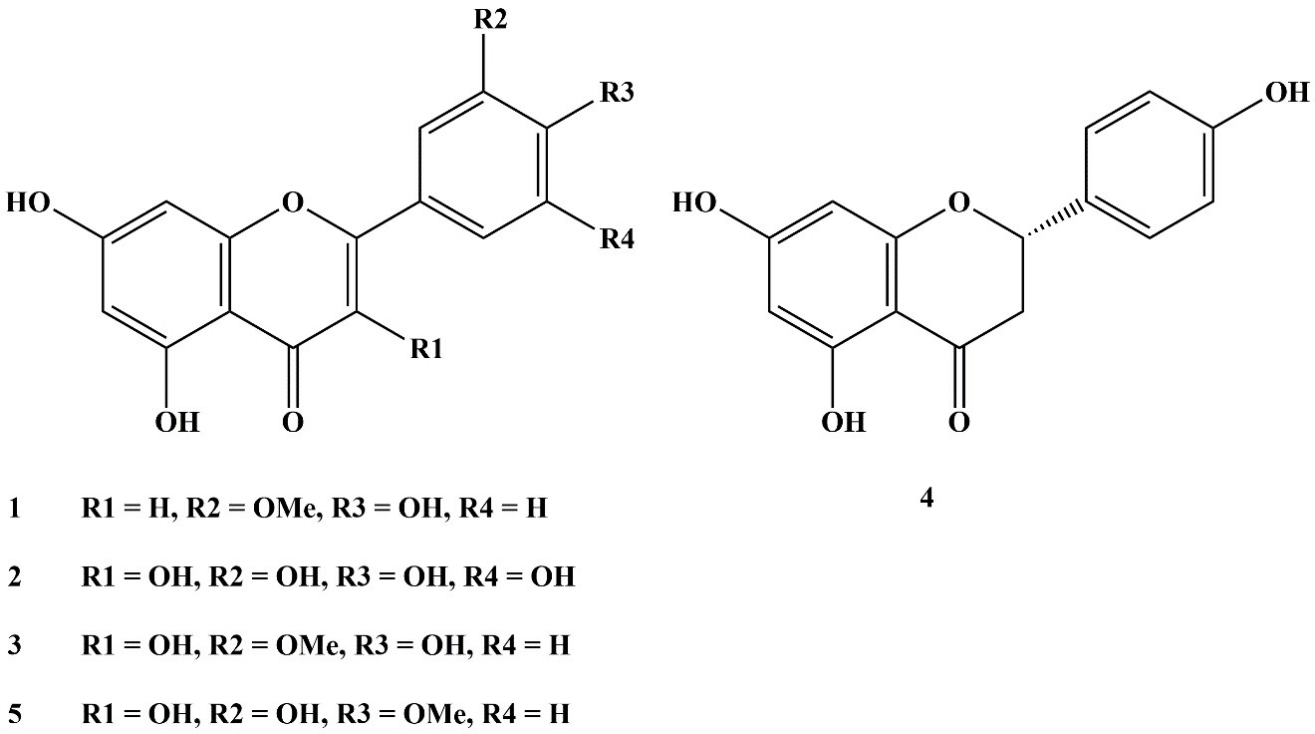
Structures of EAFAG‐isolated flavonoids.

The phytochemical fractionation of *A. graecorum* ethyl acetate fraction resulted in the isolation of five flavonoids. The structures of isolated phytochemicals were identified based on data obtained from comparison with previously reported spectral data and TLC comparisons with authentic samples. Consequently, isolated flavonoids (Figure 1) were identified as chrysoeriol (**1**),^[42]^ myricetin (**2**),^[43]^ isorhamnetin (**3**),^[44]^ naringenin (**4**),^[45]^ and tamarixetin (**5**).^[46]^

### 2.2 Inhibitory Activity Assay

**Figure 2:**
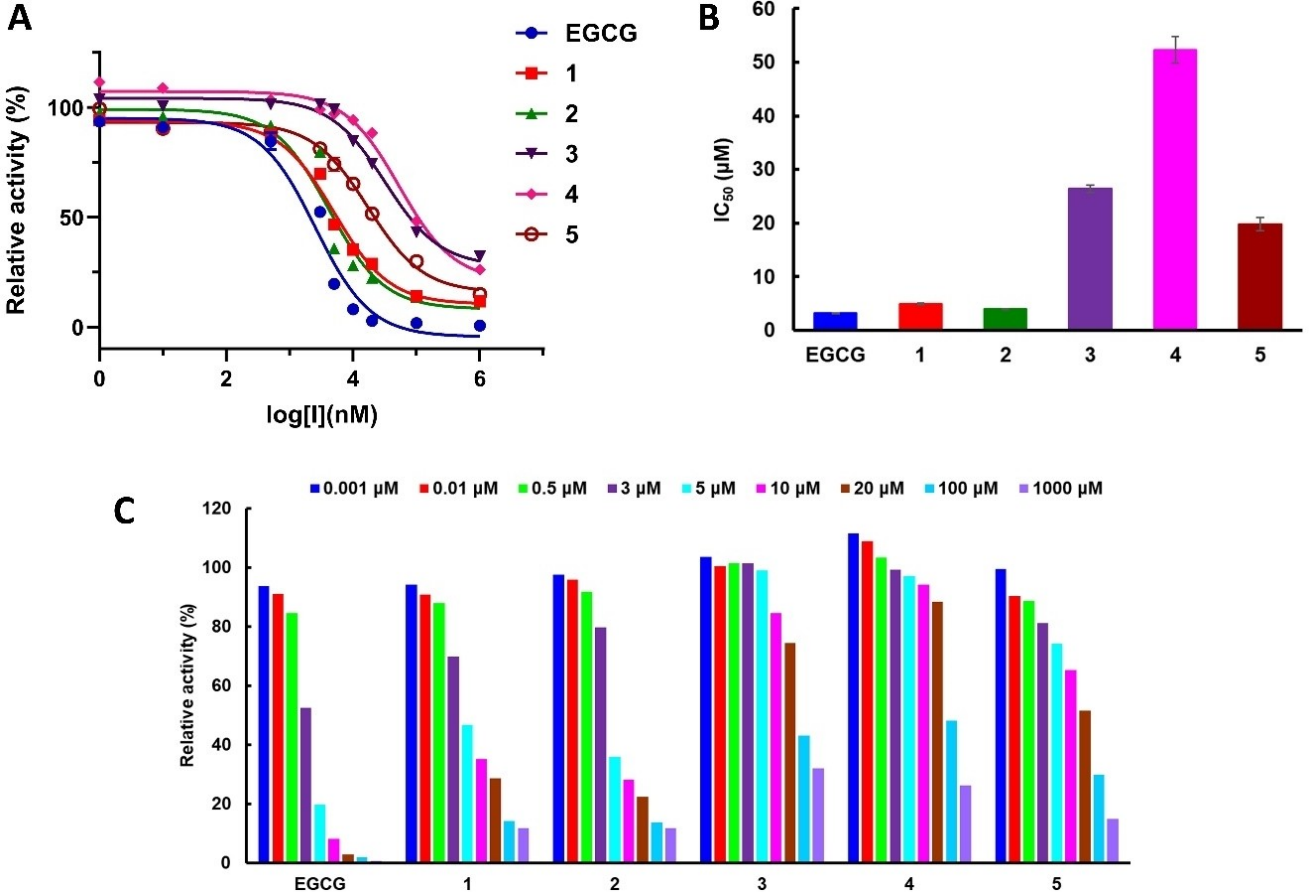
Inhibitory efficacy of drugs against β‐glucuronidase, (A) The dose‐dependent inhibition curves of investigated drugs on β‐glucuronidase activity, (B) IC_50_ values of various inhibitors in this study, and (C) different concentrations employed in assessing relative activities investigated inhibitors against β‐glucuronidase.

Given the pivotal involvement of our target enzyme in drug metabolism and its linkage to the gastrointestinal side effect of diarrhea that can occur with the cancer drug irinotecan, the quest for potent inhibitors has garnered increasing interest.^[7]^ In this study, we evaluated the inhibitory activity of chrysoeriol, myricetin, isorhamnetin, naringenin, and tamarixetin, isolated from *A. graecorum* against β‐glucuronidase, and the findings are illustrated in Figure 2 and summarized in Table 1. The isolated flavonoids exhibited dose‐dependent inhibition of β‐glucuronidase′s ability to hydrolyze the substrate PNPG, as shown in the concentration‐response curves in Figure 2A. Among the tested flavonoids, myricetin exhibited the best inhibition efficacy, indicated by its lowest IC_50_ value of 3.95±0.04 μM, followed by chrysoeriol with an IC_50_ value of 4.94±0.11 μM (Table 1 and Figure 2B). In contrast, isorhamnetin, naringenin, and tamarixetin demonstrated relatively weaker inhibition activity with IC_50_ values of 26.43±0.72 μM, 52.33±2.44 μM, and 19.85±1.23 μM, respectively. The inhibitory activity of myricetin and chrysoeriol was further proved by inhibition rate values of 92.59±0.56% and 87.97±0.65%, respectively. Interestingly, the positive control drug exhibited a comparable IC_50_ value of 3.26±0.05 μM and a high inhibition rate of 96.14±0.54%, confirming the efficiency of the assay. These results suggest that myricetin and chrysoeriol have promising potential as β‐glucuronidase inhibitors, warranting further investigation into their therapeutic applications. As represented in Figure 2C, a series of different concentrations was employed to assess the relative activities of investigated drugs against β‐glucuronidase. Remarkably, all investigated flavonoids exhibited discernible concentration‐dependent inhibition activity on β‐glucuronidase. In addition, a notable reduction in the percent of relative activity was detected with increasing inhibitor dose during the assays, affirming the inhibiton efficacy of tested flavonoids against the target enzyme.

**Table 1:** The results of the *in vitro* inhibition assay of tested flavonoids against β‐glucuronidase.

	Inhibition of flavonoids against β‐glucuronidase	
	Inhibition rate 10 μM (%)	IC_50_ (μM)
chrysoeriol (**1**)	87.97±0.65	4.94±0.11
myricetin (**2**)	92.59±0.56	3.95±0.04
isorhamnetin (**3**)	73.87±1.09	26.43±0.72
naringenin (**4**)	56.05±2.5	52.33±2.44
tamarixetin (**5**)	80.04±1.71	19.85±1.23
EGCG	96.14±0.54	3.26±0.05

### 2.3 Inhibition Kinetics

**Figure 3:**
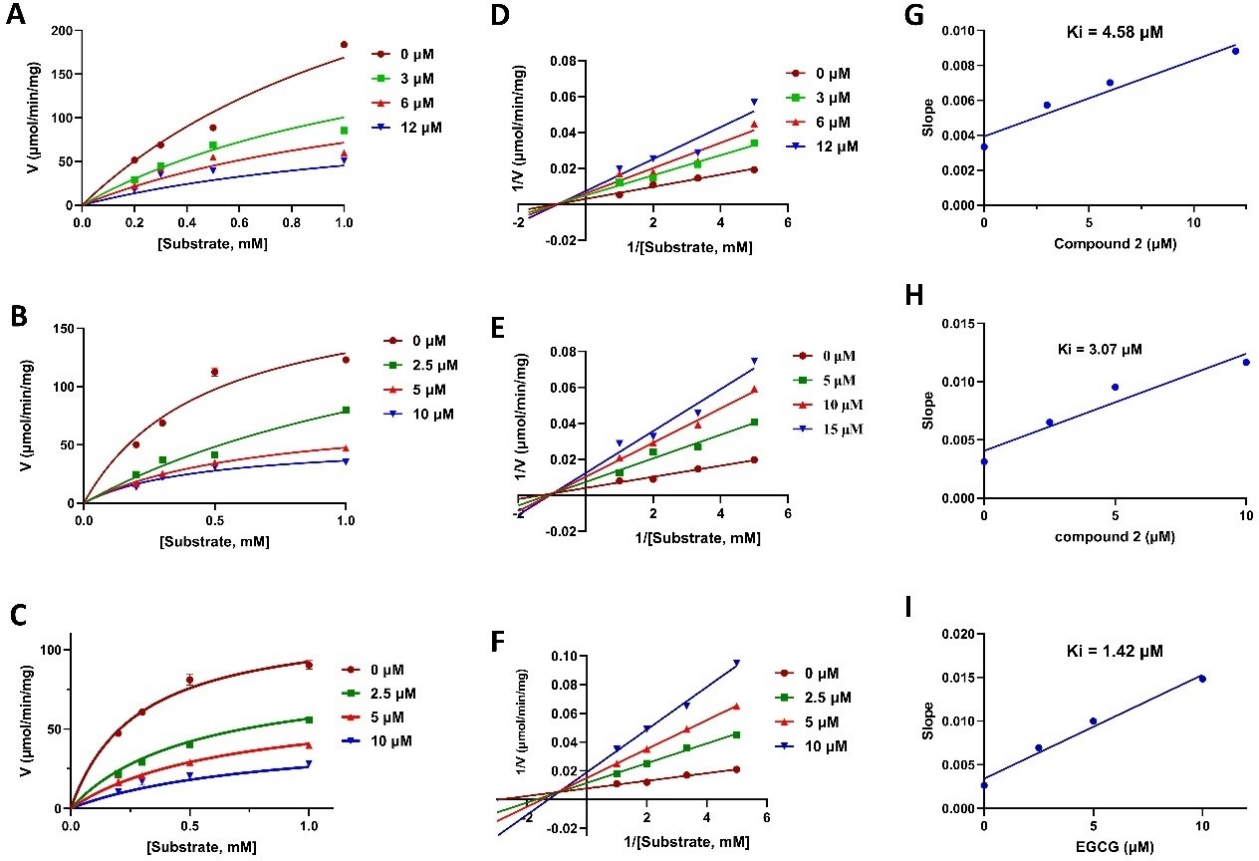
(A–C) Plots of Michaelis–Menten of chrysoeriol, myricetin, and EGCG against β‐glucuronidase, respectively. (D‐F) Lineweaver‐Burk plots of chrysoeriol, myricetin, and EGCG against β‐glucuronidase, respectively. (G‐I) inhibitors concentrations versus Lineweaver‐Burk plot slopes.

Given their highest inhibitory activities, chrysoeriol and myricetin were selected for further investigation of their enzyme kinetic analyses. By delving into the enzyme kinetics of chrysoeriol and myricetin, we aim to figure out their inhibition mechanisms and their potential as therapeutic agents for regulating the activity of the target enzyme. A series of inhibition kinetic investgations was subsequently conducted by varying substrate and inhibitor concentrations. The Michaelis‐Menten kinetics analysis revealed a dose‐dependent inhibition pattern for chrysoeriol, myricetin, and EGCG against β‐glucuronidase, as illustrated in Figure 3A–C. These findings indicate that the flavonoids may have inhibitory effects on β‐glucuronidase enzyme activity, possibly by disrupting its catalytic mechanism. These results support further investigation into the potential utility of chrysoeriol and myricetin as effective β‐glucuronidase inhibitors.

To elucidate the inhibition mechanisms and categorize the flavonoid inhibition profiles, Lineweaver–Burk plots were generated (Figure 3D–F). Notably, the EGCG plot displayed line intersections in the second quadrant, revealing a mixed mode of inhibition against the target enzyme. In contrast, Lineweaver‐Burk plots revealed that chrysoeriol and myricetin inhibit β‐glucuronidase through a noncompetitive mechanism. This suggests that these compounds bind to a site on the enzyme that is distinct from the substrate binding site. Furthermore, the estimated K_i_ values for chrysoeriol, myricetin, and EGCG were determined to be 4.58, 3.07, and 1.42 μM, respectively, as illustrated in the slope plots in Figure 3G–I. The variation in K_i_ values among tested compounds and the reference drug is mainly because of the structural variations and the inhibition modes. However, the values of Ki are in line with the values of IC_50_. These findings underscore the potent inhibitory effects of these two flavonoids from *A. graecorum* on β‐glucuronidase activity.

### 2.4 Molecular Docking

**Figure 4:**
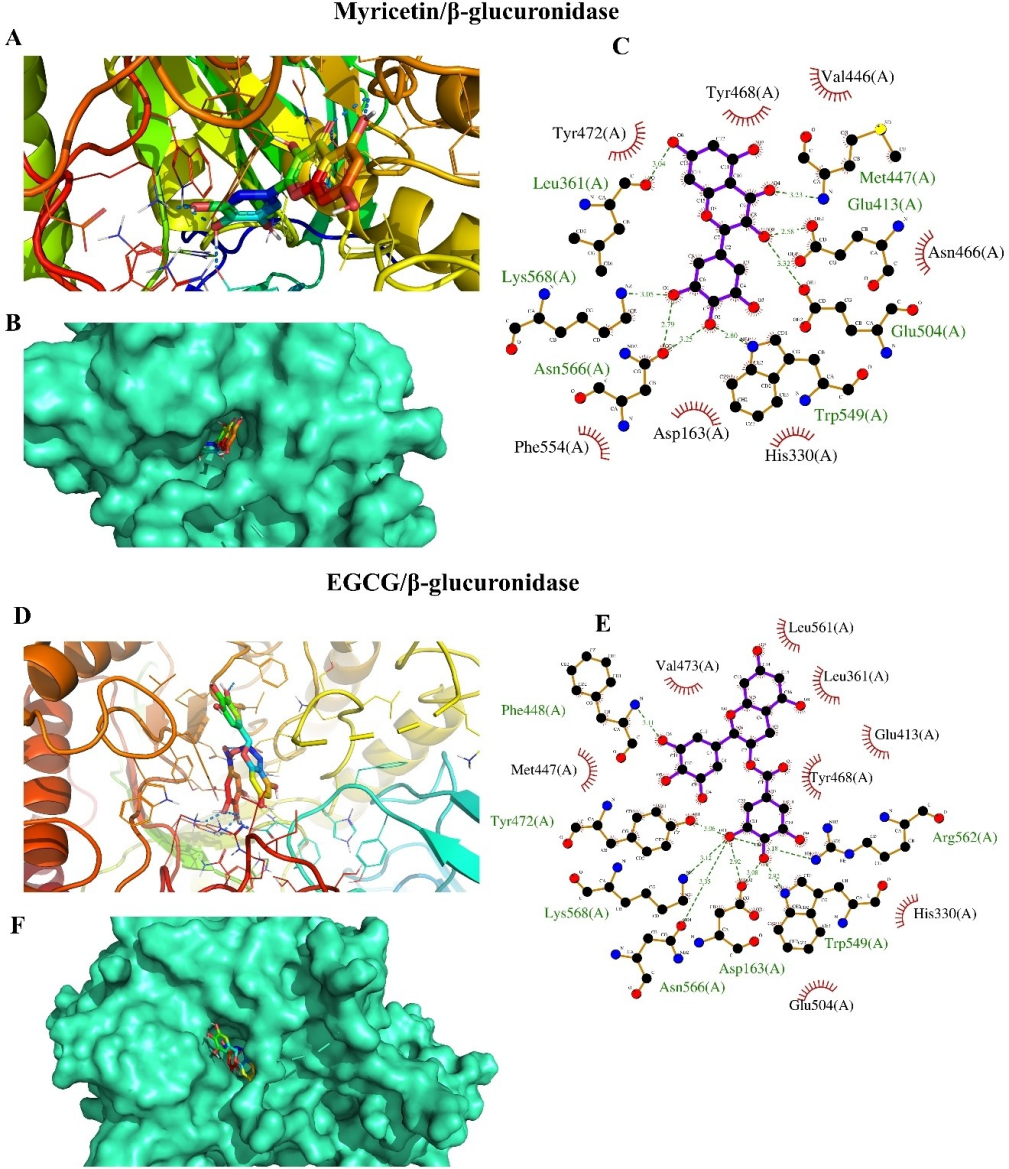
The results of docking analysis of myricetin and EGCG against β‐glucuronidase, (A, D) position of tested flavonoids within the active site of the target enzyme, (B, F) A surface representation of the active site occupied by proposed inhibitors, (C, E) residues involved in hydrophobic and polar interactions with investgated ligands.

**Figure 5:**
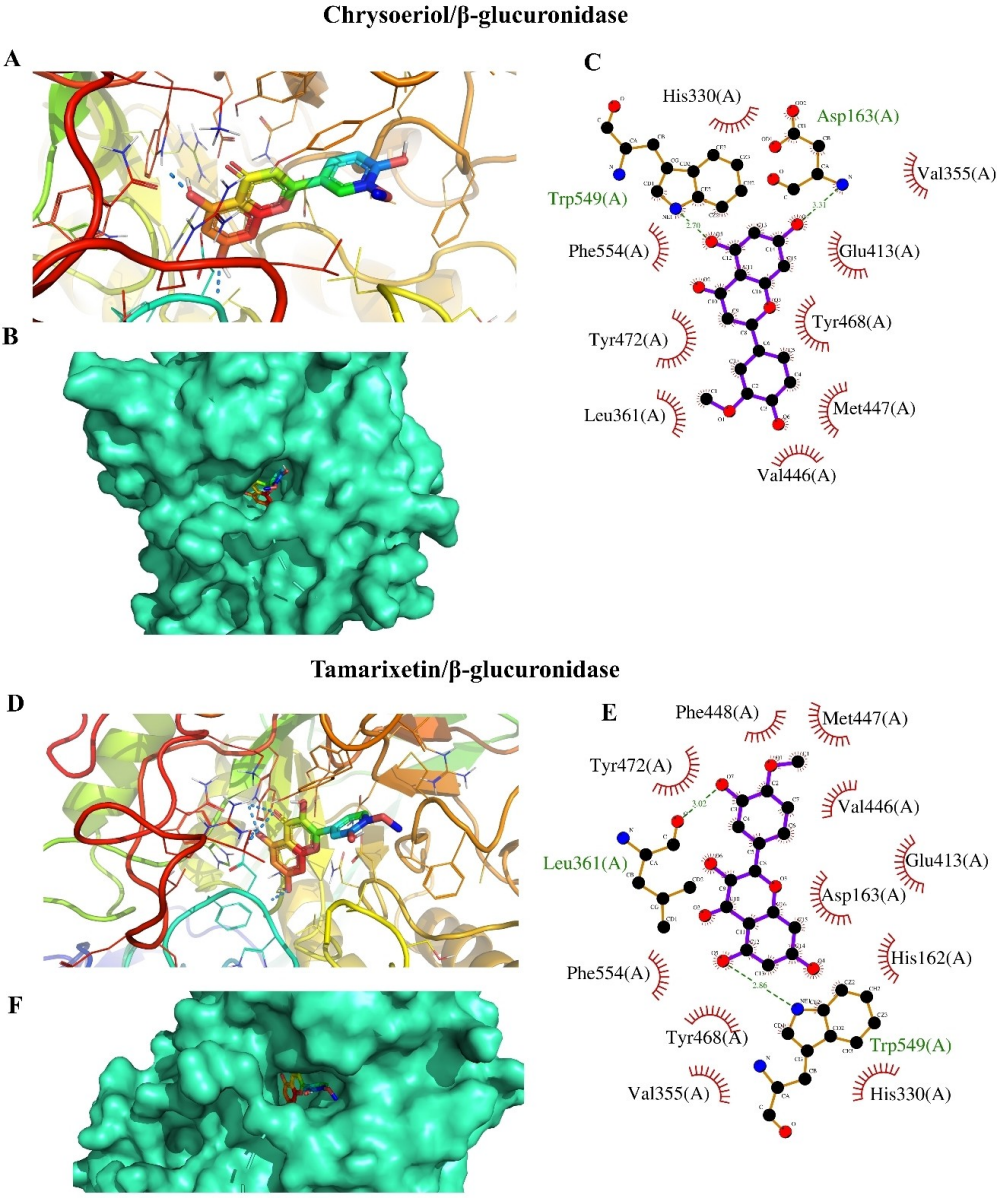
Results of docking assessments of chrysoeriol and tamarixetin against β‐glucuronidase, (A, D) position of tested flavonoids within the binding site of the target enzyme, (B, F) A surface representation of the active site of β‐glucuronidase occupied by the proposed inhibitors, (C, E) polar and hydrophobic interacting residues with investigated drugs.

**Figure 6:**
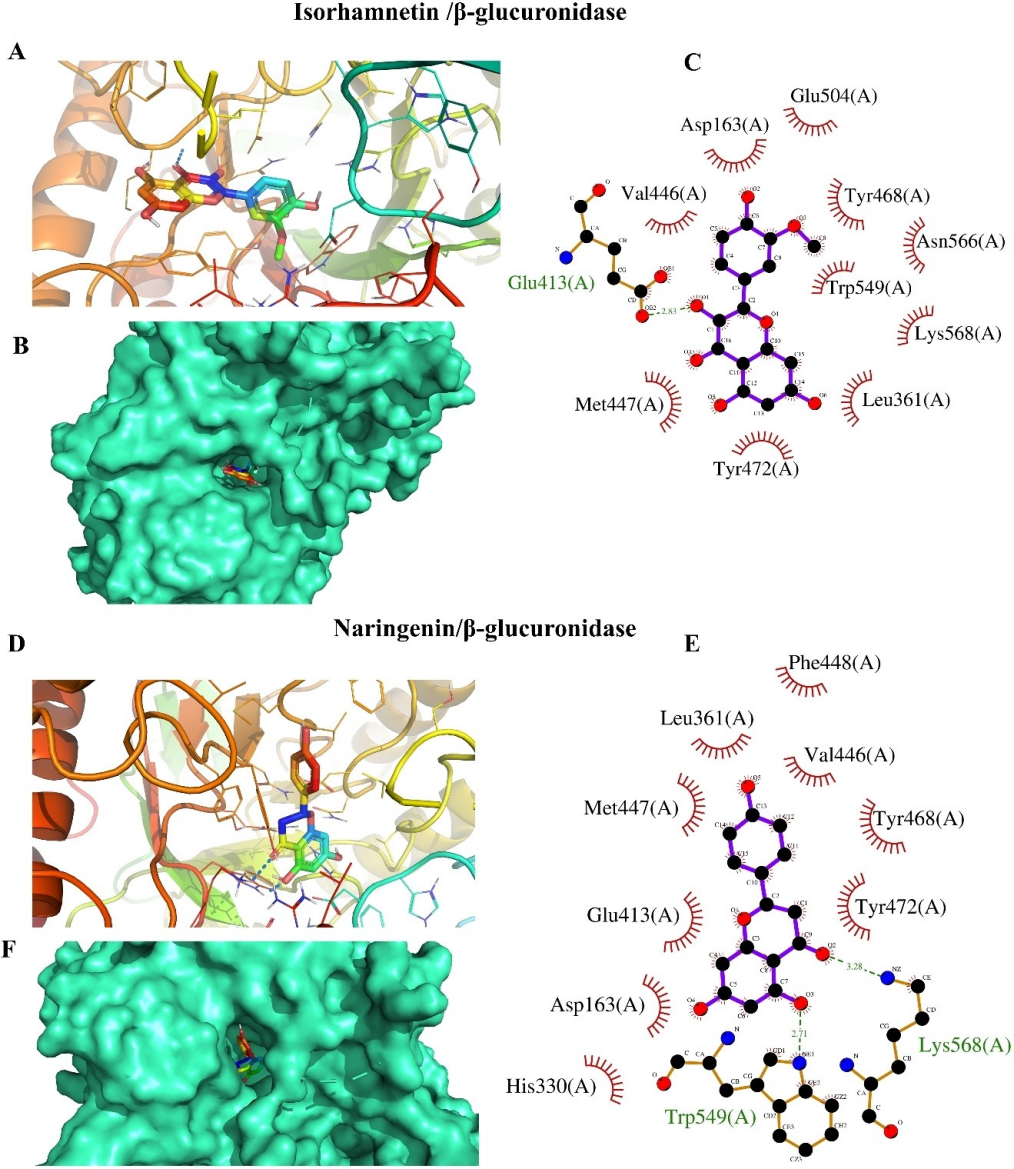
Results of docking calculations of isorhamnetin and naringenin against β‐glucuronidase, (A, D) position of tested flavonoids in the binding site of the studied enzyme, (B, F) A surface representation of the active site occupied by proposed inhibitors, (C, E) polar and hydrophobic interacting residues with flavonoids.

Molecular docking simulation was employed to study the binding modes of *A. graecorum* flavonoids with β‐glucuronidase. As shown in Table 2, chrysoeriol, myricetin, isorhamnetin, naringenin, and tamarixetin exhibited promising binding affinities, indicating strong interactions with β‐glucuronidase. The low binding affinities observed for the flavonoids suggest their potential to inhibit β‐glucuronidase. Molecular docking simulations were conducted to identify the most favorable binding orientations of these compounds within the enzyme′s active site. The results, depicted in Figures 4, 5, and 6, highlight the specific interactions between the flavonoids and the key amino acid residues in the binding pocket. These interactions involve both polar and hydrophobic forces, contributing to the overall binding affinity. Notably, all tested drugs successfully docked into identical binding sites occupied by the positive control drug, suggesting their ability to inhibit its activity potentially. Interestingly, the highest number of polar interactions was detected with myricetin and EGCG (Figure 4). This finding is of significant interest as it elucidates the mechanism behind the potent activity of myricetin. This outcome is in line with the results of the experimental inhibition assay. Additionally, chrysoeriol displayed the highest binding affinity among tested drugs, confirming its activity as a β‐glucuronidase inhibitor (Figure 5). The high extent of hydrophobic interactions in the binding profile of chrysoeriol, tamarixetin, and isorhamnetin suggests a hydrophobic binding mode for these drugs. In addition, the hydrophobic binding profiles of all tested flavonoids, except isorhamnetin (Figure 6), revealed the presence of phenylalanine residues (Phe 448 and Phe 554), which are known to participate in energetically favored π‐π interactions. These outcomes highlight the diverse modes of interaction among isolated phytochemicals and β‐glucuronidase, indicating potential variation in their mechanism of the target enzyme inhibition. The existence of important key residues in the binding profiles of these flavonoids further suggests their potential inhibitory mechanisms against β‐glucuronidase.[^1^, ^7^] Therefore, the outcomes of the docking evaluations demonstrated a degree of preference for the inhibition efficacy of chrysoeriol and myricetin against β‐glucuronidase. Consequently, further assessment of these interactions would assist in the development and design of new therapeutic drugs against β‐glucuronidase, which could be beneficial in the remedies of a range of medical conditions.

**Table 2:** The results of docking calculations β‐glucuronidase with tested flavonoids.

	Binding affinity (kcal/mol)	Polar interactions	Hydrophobic interactions
chrysoeriol (**1**)	−8.9	Asp163 and Trp549	Leu361, Glu413, His330, Met447, Val446, Tyr468, Val355, Tyr472, and Phe554
myricetin (**2**)	−8.8	Leu364, Met447, Glu504, Asn566, Glu413, Trp549, and Lys568	His330, Val446, Tyr468, Tyr472, Asp163, Asn466, and Phe554
isorhamnetin (**3**)	−8.5	Glu413	Asp163, Leu361, Met447, Val446, Tyr472, Tyr468, Trp549, Glu504, Asn566, and Lys568
naringenin (**4**)	−8.3	Trp549 and Lys568	Asp163, Leu361, His330, Glu413, Met446, Val446, Tyr468, Phe448, and Tyr472
tamarixetin (**5**)	−8.6	Leu361 and Trp549	His162, His330, Glu413, Asp163, Phe448, Val446, Tyr472, Val355, Met447, Tyr468, and Phe554
EGCG (reference drug)	−8.9	Phe448, Asp163, Tyr472, Trp549, Lys568, Asn566, and Arg562	Glu413, Tyr468, Met447, His330, Leu561, Leu361, Glu504, and Val473

### 2.5 Molecular Dynamics Simulations

In this analysis, we present the outcomes of our MD simulations aimed at exploring the dynamic behaviors and stabilities of β‐glucuronidase‐flavonoids complexes. MD simulations serve as a robust tool for elucidating the temporal evolution and structural dynamics of interactions between drugs and enzymes.[^16^, ^47^] Through these simulations, we obtained in‐depth insights into the stabilities, flexibilities, and conformational variations of complexes formed by *A. graecorum* flavonoids and β‐glucuronidase under simulated environmental conditions over time. Thus, to assess the compatibility of our flavonoids and the binding site of β‐glucuronidase, we performed MD simulations using the GROMACS package for a duration of 30 ns. The simulations centered on the drug‐protein complexes identified through docking analysis to have the most favorable binding affinities. Subsequently, an in‐depth analysis of MD trajectories of unbound β‐glucuronidase and different complexes was conducted, with a focus on crucial parameters including root mean square deviations (RMSD), radius of gyration (Rg), root mean square fluctuations (RMSF), interaction energies, solvent accessible surface area (SASA), MMPBSA, hydrogen bonding profiles, and potential energy landscape (PEL).

#### 2.5.1 Structural Stability and Dynamic Properties

**Figure 7:**
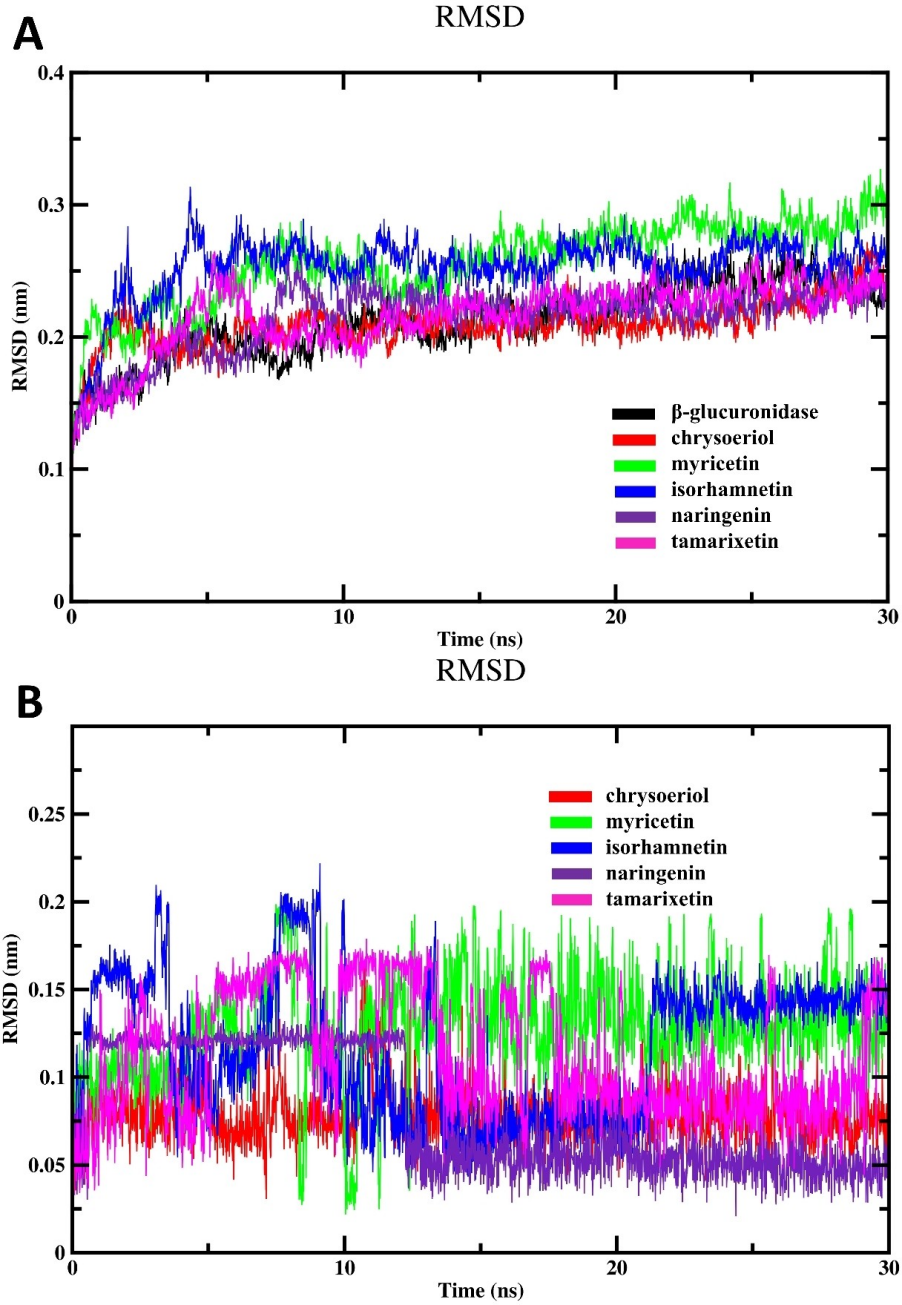
MD simulation results; (A) Backbone RMSD of unbound enzyme and different flavonoid complexes and (B) RMSD of tested flavonoids.

The evaluation of RMSD provides critical insights into the stabilities and conformational changes of biological macromolecules throughout MD simulations.^[19]^ Accordingly, we analyzed the deviation of atomic positions, specifically focusing on backbone RMSD values, across different complexes relative to the free enzyme, serving as the reference conformation throughout the simulation span. As shown in Figure 7A, it is evident that during the equilibration phase, both the free enzyme and different complexes showed a progressive rise in their backbone RMSD profile. Then, these RMSD profiles stabilized and maintained a fluctuating pattern throughout the simulation duration. Interestingly, myricetin depicted the highest RMSD values after reaching equilibrium, signifying considerable conformational changes or structural flexibility within the enzyme‐drug complex throughout the simulation process. In a similar vein, these variations might be explained by the dynamic interactions between myricetin and β‐glucuronidase binding site. This suggests that myricetin can adjust its binding conformation to optimize its inhibitory effect. These outcomes align well with the results of *in vitro* assay. The lowest RMSD values observed for chrysoeriol suggest a stable binding conformation within the enzyme′s active site. This stability is further supported by the minimal structural fluctuations observed for chrysoeriol during the simulations. This stability allows for sustained and effective binding interactions, leading to high inhibitory activity. Nevertheless, the RMSD fluctuations observed for the remaining flavonoids fall within the typical range for stable complexes, indicating relatively stable binding configurations with β‐glucuronidase throughout the MD simulation period.

To independently assess the conformational changes and inherent motion of the flavonoid, we calculated their RMSD values throughout the simulations (Figure 7B). The analysis involved aligning each trajectory frame to an initial geometry of the flavonoid compound. Then, the average displacement of the atomic coordinates of the ligand was computed relative to this reference geometry. Consequently, this approach allows us to assess how much the flavonoid deviates from its initial geometry over the simulation span, providing crucial information into the dynamics and stability of each drug. Such analysis is crucial in the field of drug design, where understanding a drug molecule′s behavior is essential. As shown in Figure 7B, the assessment of RMSD for different flavonoids exhibited a significant variation in their RMSD profile. Interestingly, only myricetin and chrysoeriol displayed typical fluctuation patterns in their relative RMSD profiles (drug‐to‐drug), which aligns with their inhibition efficacy. This outcome indicated that these drugs adopt stable conformations during the simulation, potentially indicating strong and consistent interactions with the binding site. The high RMSD profile observed for myricetin could suggest its ability to undergo specific deviations from the initial conformation, enabling it to better accommodate the binding site of the enzyme. Conversely, the observation that the lowest drug‐to‐drug RMSD values were detected for naringenin, the less active drug with the highest IC_50_, implies that the structural rigidity of this compound may limit its ability to maintain a stable conformation over time.

**Figure 8:**
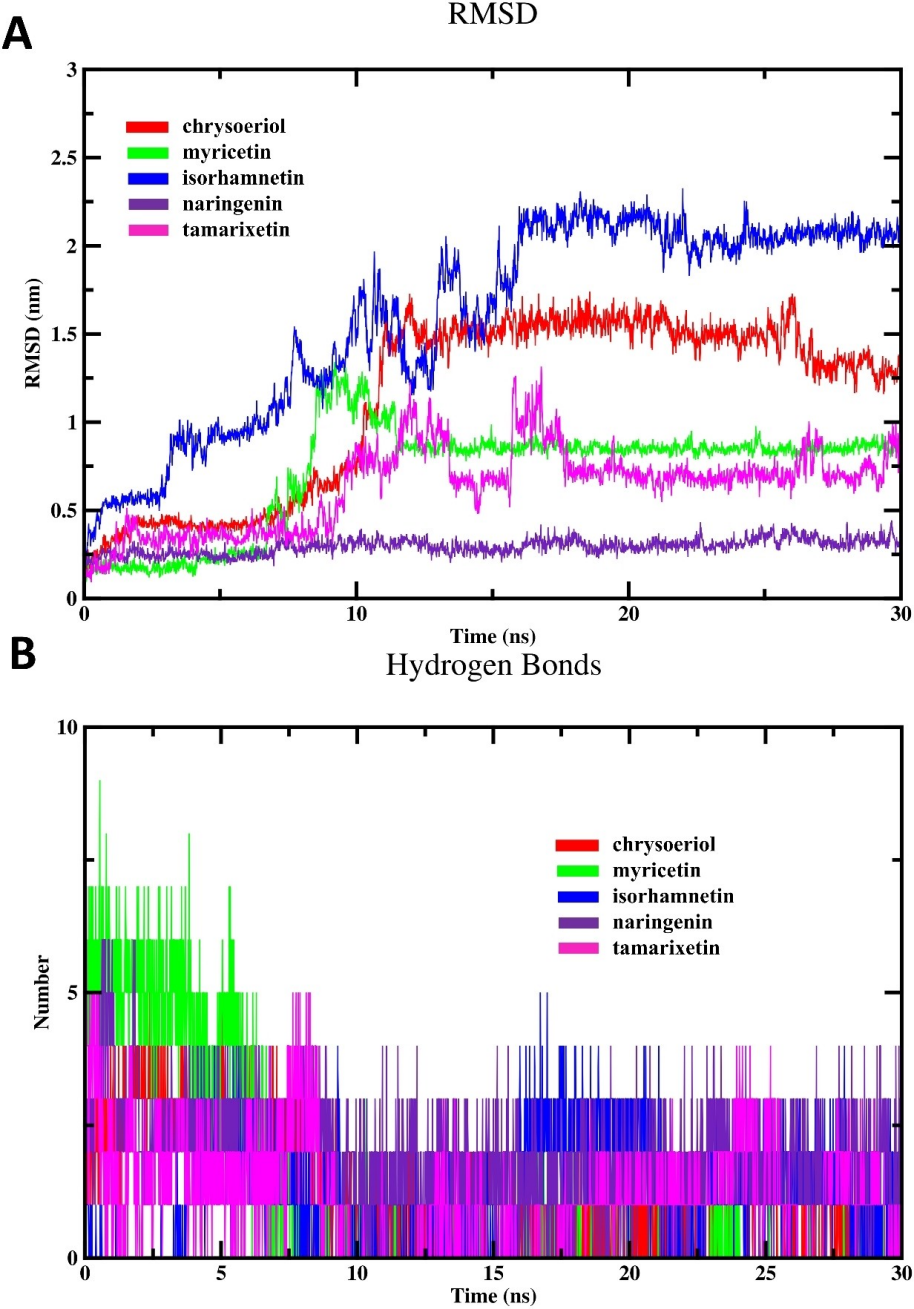
Results of MD simulation; (A) Backbone RMSD of different complexes and (D) Hydrogen bonding of flavonoids‐ β‐glucuronidase complexes.

The RMSD profile using both the enzyme and the investigated flavonoid coordinates can provide crucial information into the structural stability and conformational changes of the drug molecule relative to the protein backbone. This approach allows us to assess how the drug interacts with the protein backbone and how its conformation evolves throughout the simulation, providing valuable information about the dynamics of the drug‐protein complex. As represented in Figure 8A, the lower RMSD values of naringenin complex indicate that this flavonoid remains largely unchanged and does not significantly interact or bind with the binding cavity of β‐glucuronidase. The lack of significant structural changes or deviations from the initial conformation could indicate that the drug does not effectively engage with the enzyme or induce any conformational changes necessary for inhibitory activity. Thus, the low RMSD value may reflect the absence of stable binding between the flavonoid and β‐glucuronidase, consistent with its inactivity. In contrast, chrysoeriol and myricetin exhibited an initial upward trend in RMSD profile during the initial 12 ns of the simulation period, after which they stabilized and displayed normal fluctuations. This observed behavior aligns with the inhibitory activities demonstrated by these drugs against β‐glucuronidase.

Subsequently, we examined the binding properties of the tested drug within the binding cavity of β‐glucuronidase by assessing the hydrogen bonding profiles of different systems, as depicted in Figure 8B. The analysis of hydrogen bonds demonstrated a robust and consistent existence of hydrogen bonding, corroborating the results obtained from docking investigations. Our findings from MD analysis revealed that myricetin formed a higher number of hydrogen bonds compared to other compounds. This observation, coupled with the high binding affinity and the strongest inhibitory potency in enzyme inhibition assays, suggests a potential link with hydrogen bonding. From these observations, it can be inferred that myricetin likely forms stable interactions with the binding site, as evidenced by its numerous hydrogen bonds.

**Figure 9:**
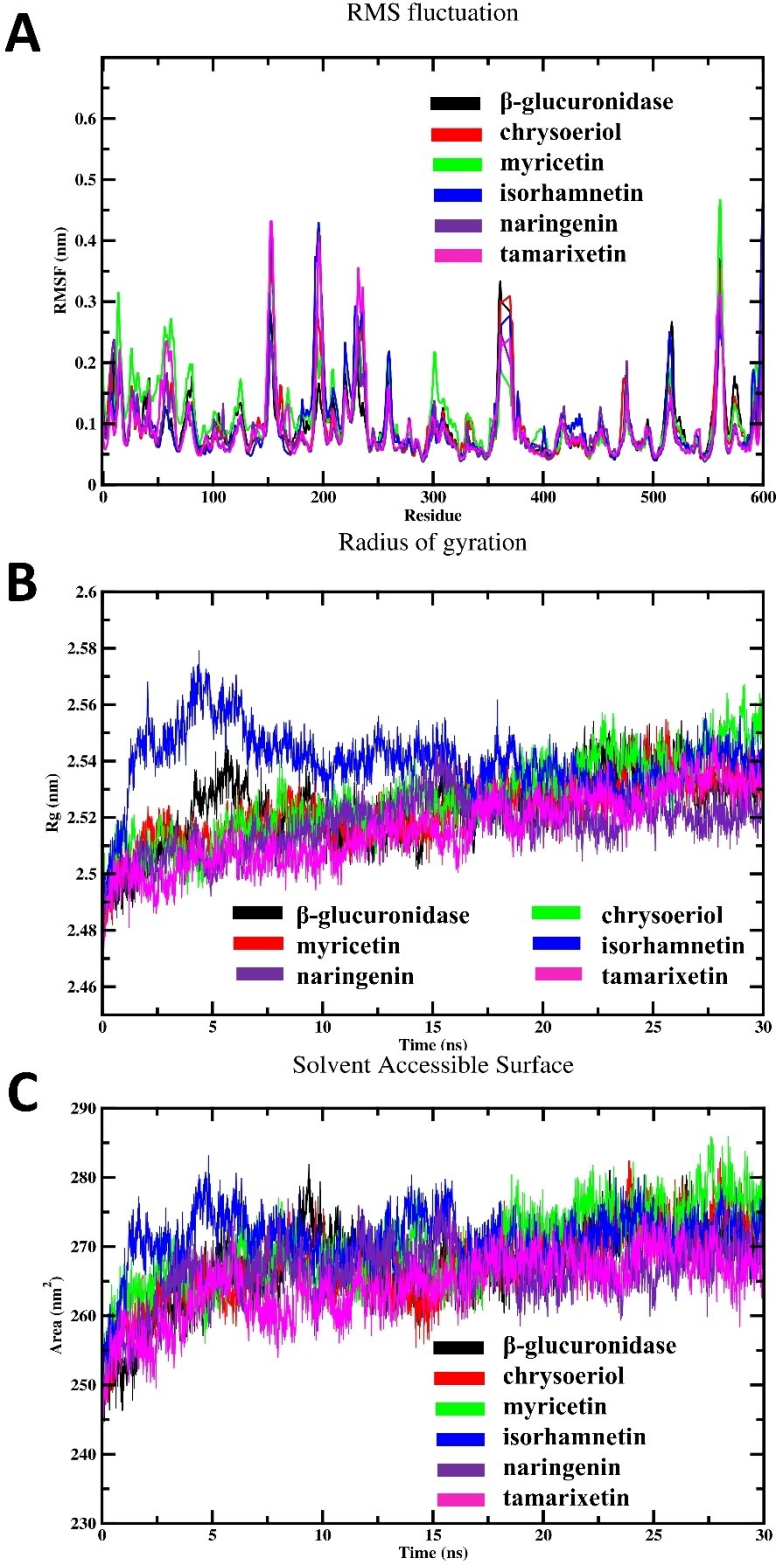
The results of MD simulation; (A) Time‐averaged RMSF profile of unbound enzyme and different tested inhibitor complexes, (B) The Rg profiles of of free enzyme and various inhibitor complexes, and (C) SASA profiles of investigated systems.

Next, we computed the RMSF profile for the free enzyme and different flavonoid complexes, targeting local protein mobility assessments, as depicted in Figure 9A. This plot illustrates RMSF values plotted against the number of residues. As anticipated, the RMSF values of β‐glucuronidase‐flavonoids complexes exhibited similar patterns to that of free enzyme, suggesting a negligible alteration in the movement of binding site residues. Based on our observations, it can be inferred that the tested drugs likely do not cause substantial alterations to the conformation of the target enzyme′s binding pocket. Interestingly, myricetin exhibits more fluctuation peaks in its RMSF profile, suggesting that this compound may interact with different regions of the enzyme or induce varying degrees of flexibility in the enzyme structure with respect to other drugs.

The investigation of the dynamics and stability of different complexes was facilitated through the examination of Rg values.^[48]^ These metrics serve as indicators of enzyme compactness, portraying alterations in folding, unfolding, and conformational transitions throughout the simulation period.[^21^, ^22^, ^48^] The Rg profile of free β‐glucuronidase and different inhibitor complexes were calculated and are represented in Figure 9B. For myricetin, despite being a potent inhibitor with a low IC_50_ value, its higher Rg values during the last 10 ns of the simulation suggest increased molecular flexibility or expansion. Conversely, naringenin demonstrated lower Rg values, indicating a higher compactness and minimum enery conformation. The higher Rg values of myricetin and chrysoeriol complexed with the enzyme compared to the unbound β‐glucuronidase suggest an expansion or increased flexibility of the enzyme‐drug complexes. This could indicate that myricetin and chrysoeriol induce structural changes or interactions within the binding site.

SASA is a widely used and dependable metric for analyzing the interactions between enzymes and solvents.[^16^, ^25^] By measuring SASA, researchers gain valuable insights into potential conformational alterations that enzymes undergo during the binding process and can assess how accessible the enzyme is to the solvent.[^13^, ^16^, ^47^] Figure 9C depicts the detected SASA fluctuations in free enzyme and different studied inhibitor complexes. Interestingly, myricetin and chrysoeriol showed higher SASA values after equilibration during the second half of the simulation time. The higher SASA values of myricetin and chrysoeriol complexed with the enzyme compared to other studied drugs with higher IC_50_ values suggest that these compounds have a larger surface area exposed to the surrounding solvent when bound to the enzyme. This increased solvent accessibility may indicate that myricetin and chrysoeriol form more extended or flexible interactions with the binding pocket, resulting in a less compact or more dynamic complex. In contrast, the lower SASA detected for other studied drugs with higher IC_50_ values indicate that these compounds form more compact or less flexible complexes, affecting their inhibitory potential.

#### 2.5.2 Interaction Energies

**Figure 10:**
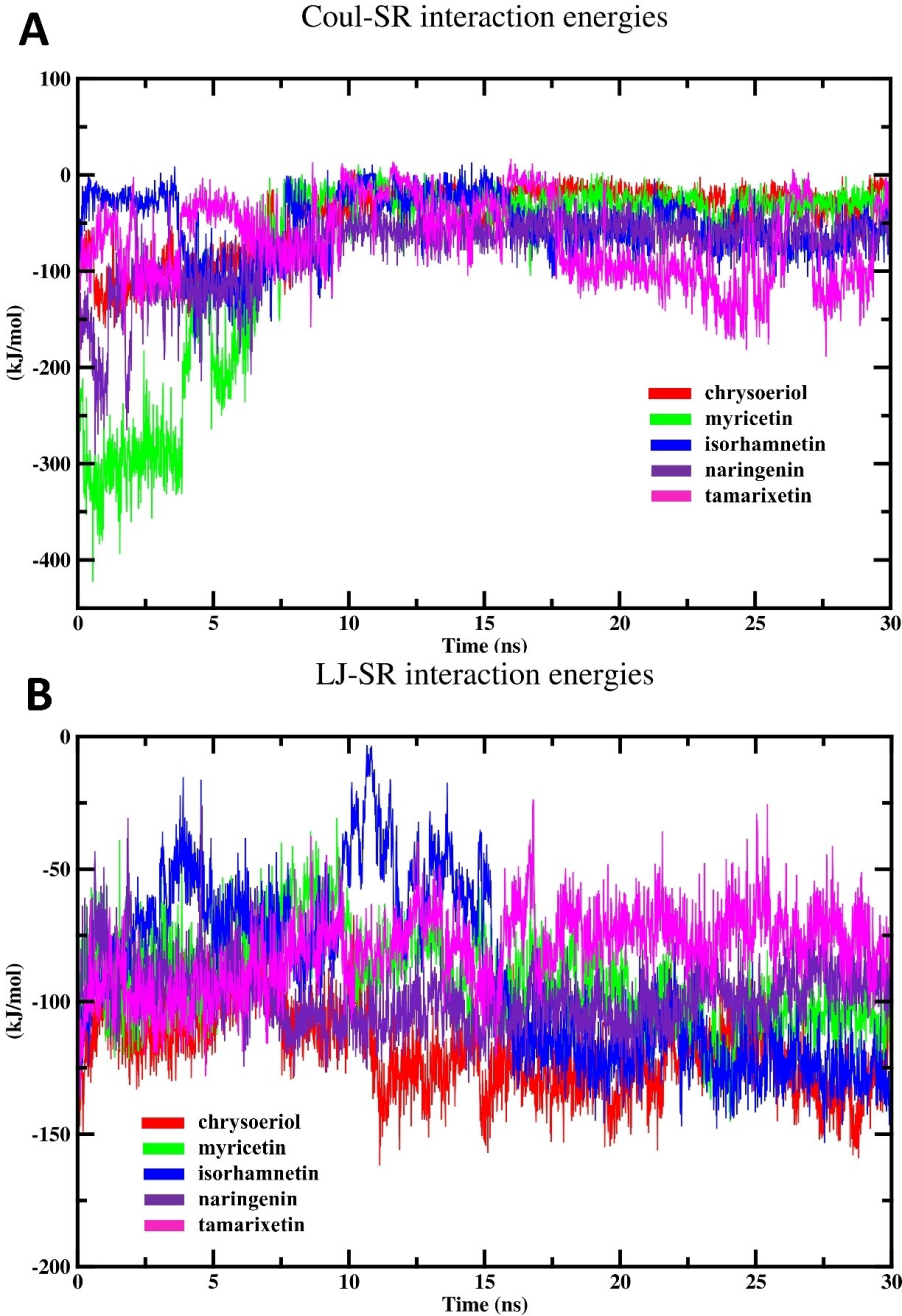
The MD simulation results; (A) Coul‐SR energy profile of different complexes and (B) LJ‐SR values of different systems.

In simulations of drug‐enzyme complexes, calculating interaction energies including short‐range Coulombic (Coul‐SR) and Lennard‐Jones (LJ‐SR) offers crucial insights into the forces governing the complex′s stability and binding affinity.[^16^, ^47^] These interactions encompass both electrostatic and van der Waals energies between flavonoids and the β‐glucuronidase. Specifically, Coul‐SR interactions capture the electrostatic attractions and repulsions between charged groups. Meanwhile, LJ‐SR interactions account for the attractive dispersion forces that promote binding, along with the repulsive forces that arise when electron clouds overlap excessively.^[49]^ The interaction energies between the target enzyme and investigated flavonoids throughout the simulation period are represented in Figure 10, specifically highlighting the Coul‐SR and LJ‐SR energies. In addition, Table 3 provides the interaction energies of various flavonoids‐β‐glucuronidase systems.

**Table 3:** The findings from the average interaction energy calculations for various studied systems.

	Coul‐SR		LJ‐SR	
	Average (kJ/mol)	RMSD (nm)	Average (kJ/mol)	RMSD (nm)
chrysoeriol	−45.213±15	35.7608	−118.616±4.3	15.4895
myricetin	−77.4139±4.4	95.8796	−91.6955±5.6	17.1215
isorhamnetin	−52.8907±5.2	32.1657	−91.1681±13	32.1747
naringenin	−76.1547±15	37.6336	−98.7023±3	11.9075
tamarixetin	−67.5386±13	42.8614	−80.9564±4.6	14.9491

Figure 10A represents the Coul‐SR values of different studied systems. This energy profile attained equilibrium at about 7.5 ns and then fluctuated normally till the simulation was completed. As shown in Table 3, myricetin showed the minimum average Coul‐SR and the highest Coul‐SR‐RMSD value, indicating robust electrostatic interactions with the binding site residues. These interactions may involve favorable charge‐charge interactions between the flavonoid and specific residues in the enzyme, contributing to its inhibitory potency. The high Coul‐SR RMSD value for myricetin indicates significant conformational changes during the simulation span. While this flexibility may lead to fluctuations in binding geometry, it could also enable myricetin to adopt multiple conformations that effectively inhibit enzyme activity. Also, myricetin may adopt a dynamic binding mode, where it transiently interacts with the enzyme in multiple orientations or conformations. This dynamic behavior allows myricetin to explore different binding poses and optimize its interactions with the enzyme, leading to effective inhibition despite fluctuations in Coulombic interaction energy. These outcomes confirm the inhibitory potency of myricetin against the target enzyme as estimated by the *in vitro* inhibitory efficacy.

The LJ‐SR interaction energies (Figure 10B) were deemed reliable indicators for predicting binding interactions within the active site. Notably, all tested drugs reached equilibrium during the second half of the simulation span. As depicted in Table 3, chrysoeriol displayed the more negative LJ‐SR value, indicating the engagement in Van der Waals interactions. These interactions involve attractive forces between the non‐polar regions of chrysoeriol and complementary residues at the binding site. Also, chrysoeriol may adopt a binding pose or conformation that allows it to maximize favorable Van der Waals interactions. This inference is in agreement with the outcomes of docking results indicating that that hydrophobic interactions play a significant role in the binding of chrysoeriol to the enzyme. In addition, the less negative average LJ‐SR energy values of tamarixetin may be influenced by its specific molecular interactions, structural features, conformational dynamics, and overall binding affinity with the enzyme. Despite this, its moderate IC_50_ value with respect to other compounds suggests that it retains a moderate degree of inhibitory potency against β‐glucuronidase.

Therefore, the observation that Coul‐SR interactions are relatively weaker compared to LJ‐SR interactions implies a predilection for Van der Waals forces over electrostatic interactions within the atoms that make up the complexes. This observation implies that hydrophobic interactions, such as dispersion forces, primarily contribute to the stabilization of certain complexes. Furthermore, the characteristic fluctuations and more negative values of both calculated interaction energies indicate minimum energy interactions between the flavonoids and the target enzyme. Consequently, the complexes exhibit a balanced combination of kinetic and energetic favorability, without a single dominant interaction type.

#### 2.5.3 MM/PBSA Analysis

The MM/PBSA results for the binding energies of isolated drugs to the target enzyme are summarized in Table 4, highlighting the van der Waals (ΔE_vdw_), electrostatic (ΔE_ele_), solvation (ΔG_solv_), gas‐phase (ΔG_gas_), and total binding free energies (ΔG_total_).

**Table 4:** The findings of MM/PBSA calculations (kcal/mol).

System	ΔE_vdw_	ΔE_ele_	ΔG_solv_	ΔG_gas_	ΔG_total_
chrysoeriol	−31.17±0.89	−15.04±2.41	25.55±0.55	−46.21±2.59	−20.65±2.65
myricetin	−32.58±0.34	−29.33±1.40	37.98±1.24	−61.91±1.74	−23.93±2.98
isorhamnetin	−23.86±0.92	−17.30±1.53	27.00±1.64	−41.16±2.45	−14.16±4.09
naringenin	−26.58±0.33	−19.96±2.06	30.67±1.01	−46.54±2.39	−15.87±3.40
tamarixetin	−22.01±0.24	−29.54±1.77	39.84±1.32	−51.55±2.01	−11.71±3.33

The MM/PBSA analysis of isolated flavonoids against β‐glucuronidase reveals varying degrees of binding affinity and stability. Among the flavonoids studied, myricetin and chrysoeriol demonstrated the most favorable binding interactions, as indicated by their significantly lower total binding free energies (ΔG_total_) of −23.93±2.98 kcal/mol and −20.65±2.65 kcal/mol, respectively. These results suggest that these two flavonoids form the most stable complexes with β‐glucuronidase. Notably, myricetin displayed the lowest IC_50_ value among the flavonoids tested, indicating its high potency as an inhibitor. Chrysoeriol also showed strong binding interactions, with substantial van der Waals and electrostatic contributions to its binding affinity. Other flavonoids, such as isorhamnetin, naringenin, and tamarixetin, exhibited higher ΔG_total_ values, indicating less favorable binding interactions.

#### 2.5.4 Principal Component Analysis (PCA) and Potential Energy Landscape (PEL)

**Figure 11:**
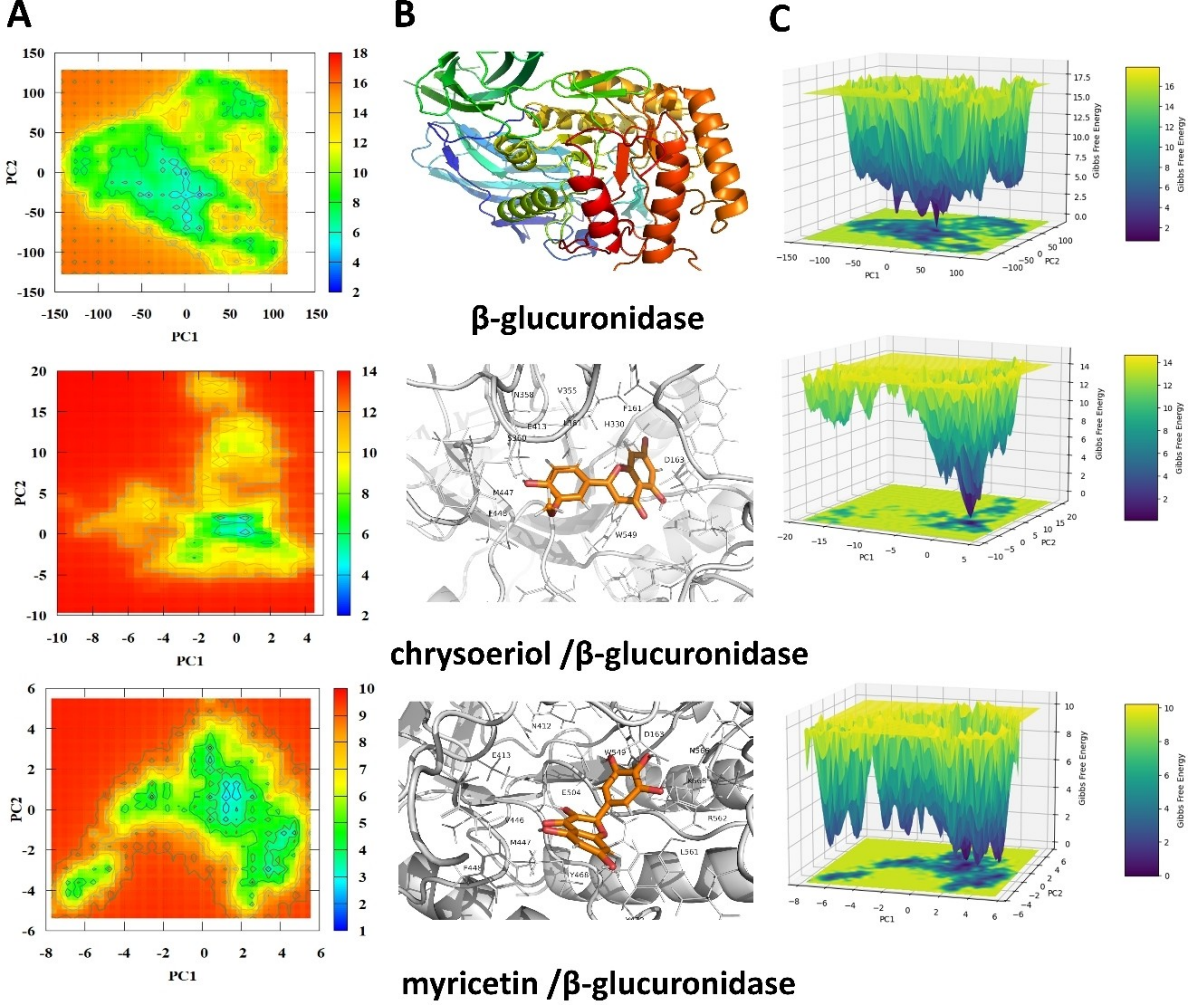
Presents the potential energy landscape (PEL) analysis of unbound β‐glucuronidase and its complexes with chrysoeriol and myricetin. Panel A shows a 2D representation of the PEL. Panel B illustrates the binding poses of these flavonoids within the β‐glucuronidase active site, highlighting key residues involved in the interactions. Panel C provides a 3D visualization of the PEL.

**Figure 12:**
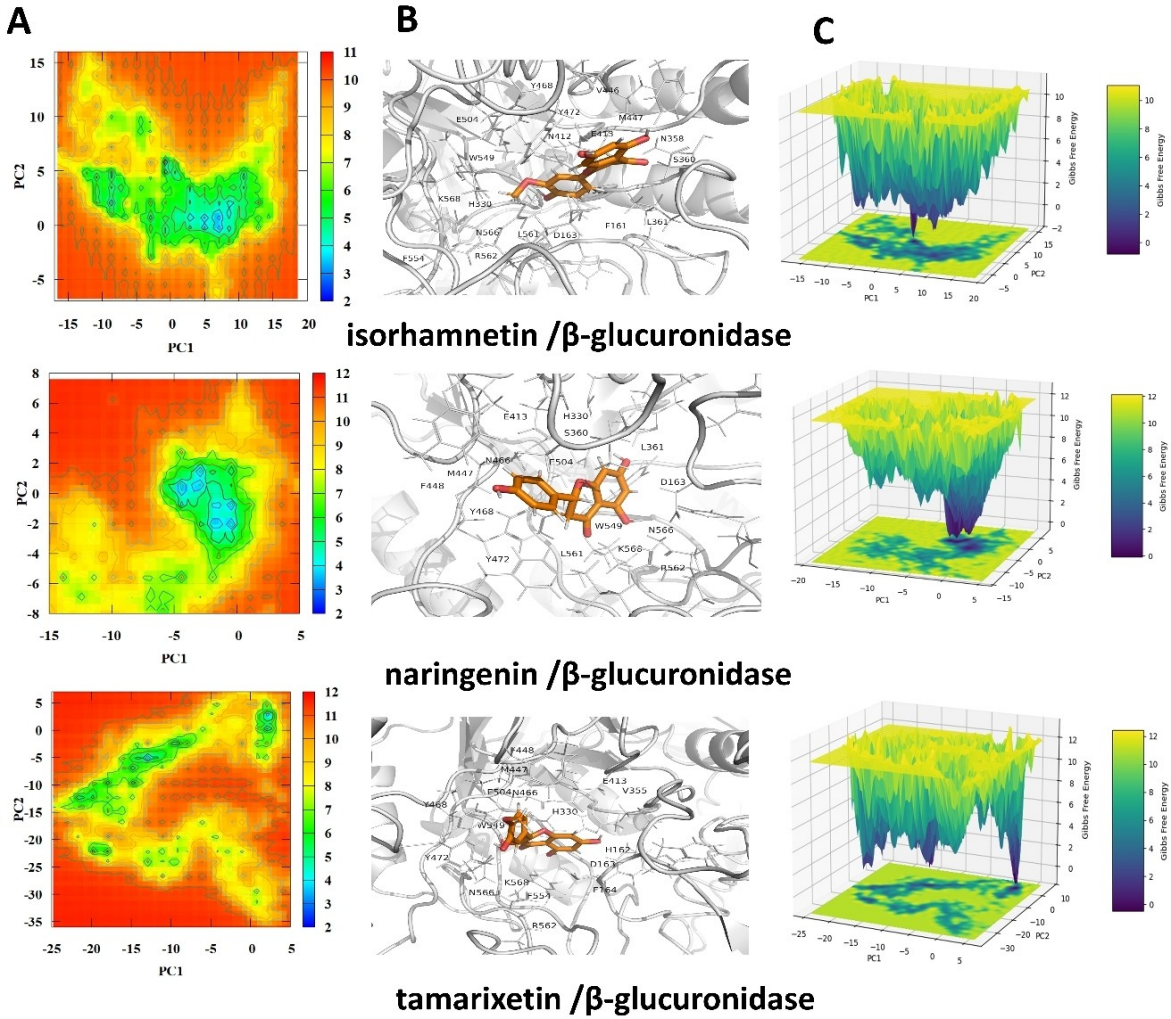
Presents the potential energy landscape (PEL) analysis of β‐glucuronidase complexes with isorhamnetin, naringenin, and tamarixetin. Panel A shows a 2D representation of the PEL. Panel B illustrates the binding poses of these flavonoids within the β‐glucuronidase active site, highlighting key residues involved in the interactions. Panel C provides a 3D visualization of the PEL.

Figures 11 and 12 present the PCA and PEL analyses for β‐glucuronidase and its complexes with various flavonoids. These analyses provide insight into the conformational stability and dynamics of the enzyme in the presence of these inhibitors. In these figures, panel A displays the 2D heat map of the free enzyme and various studied systems, panel B represents conformations corresponding to the predicted PEL energy minima, and panel C displays a 3D representation of the PEL.

The PCA results indicate that the binding of the tested drugs to β‐glucuronidase significantly impacts the conformational dynamics of the enzyme. Myricetin and chrysoeriol, which exhibited the lowest IC_50_ values, show distinct and stable binding conformations, as evidenced by the dense clustering of points in the PCA plots (Figure 11A) and the deep valleys in the PEL plots (Figure 11C). This suggests a strong and stable interaction between these flavonoids and the enzyme, corroborating their potent inhibitory effects. The unbound enzyme showed a wider range of conformational states, suggesting a more open and accessible active site. However, when bound to flavonoids, the enzyme adopted a more compact and closed conformation, potentially hindering substrate binding. This conformational change highlights the potential of these flavonoids to effectively block the enzyme′s active site.

## 3 Conclusions

To summarize, the application of chromatographic techniques on EAFAG led to the isolation of five previously unreported flavonoids in *Alhagi graecorum*. To assess the inhibition capacity of these compounds against β‐glucuronidase, an integrated approach utilizing computational and experimental techniques was employed. The experimental *in vitro* inhibitory potential of isolated phytochemicals on β‐glucuronidase was carefully investigated. Our results showed that myricetin and chrysoeriol displayed strong inhibition against β‐glucuronidase with the minimum IC_50_ values of 3.95±0.04 and 4.94±0.11 μM, respectively. Additionally, myricetin and chrysoeriol inhibited the target enzyme through noncompetitive mechanisms, with K_i_ values of 3.07 and 4.58 μM, respectively. The results of docking assessments revealed low binding affinities for tested drugs against the target enzyme with the largest number of polar interactions with myricetin and EGCG. A relatively high extent of hydrophobic interactions was detected in the binding modes of different systems. In addition, tested drugs occupied similar binging pocket to that of the positive control drug.

To gain a deeper understanding of the interactions between the flavonoids and β‐glucuronidase, we conducted extensive MD simulations. Over a 30 ns simulation timeframe, we meticulously analyzed a comprehensive set of parameters. Our comprehensive investigation showed consistent trajectories for evaluated compounds, with myricetin and chrysoeriol demonstrating notable equilibration of energy levels. Furthermore, only the drug‐to‐drug RMSD profiles of myricetin and chrysoeriol exhibited typical fluctuation patterns. These findings are in agreement with their β‐glucuronidase inhibition efficacy, as indicated by their low IC_50_ values. Furthermore, analysis of hydrogen bonding revealed robust interactions among the flavonoids and β‐glucuronidase, with myricetin exhibiting a higher extent of hydrogen bonding. The RMSF values revealed that the presence of flavonoids stabilized specific regions within the enzyme structure. Moreover, examination of Rg and SASA values suggested a reduction in the enzyme flexibility and accessibility when binds to the flavonoid inhibitors, particularly with myricetin and chrysoeriol, suggesting the stability of the different complexes. Finally, the assessment of interaction energies depicted a harmonious and stable interaction among flavonoids and the target enzyme. Myricetin displayed the lowest Coul‐SR profile and the highest Coul‐SR‐RMSD value, indicating strong electrostatic interactions. Conversely, chrysoeriol displayed a more negative LJ‐SR value, suggesting preference to Van der Waals interactions with the binding pocket of the enzyme. These results align well with the results of the experimental inhibitory activity, providing crucial insights into the inhibition modes of flavonoids from *A. graecorum* as β‐glucuronidase inhibitors. This study provides a solid base for the design and development of new β‐glucuronidase inhibitors with promising therapeutic applications.

## 4 Declarations

### 4.1 Funding

This project was funded by Researchers Supporting Project number (RSPD2024R1078), King Saud University, Riyadh, Saudi Arabia. Also, this work was carried out with the support from the project PID2023‐150717NB‐I00 from Ministerio de Ciencia, Innovacion y Universidades in Spain and the PRIES‐CM project Ref: Y2020/EMT‐6290 from the Comunidad Autónoma de Madrid. Lastly, the authors express their gratitude to the Centro de Computación Científica of the UAM (CCC‐UAM) for providing the computing time.

## Author Contributions

Conceptualization, E. M. K., A‐M. L., and S. M; methodology, E. M. K., E. M. A.; software, E. M. K. and A‐M. L. and; validation A‐M. L., S. M. and E. M. K.; formal analysis, A‐M. L., S. M. and E. M. K.; investigation, S. M., E. M. A. and E. M. K.; resources S. M., E. M. A. and A‐M. L.; data curation, E. M. K., and A‐M. L.; writing–original draft preparation, E. M. K.; writing–review and editing, E. M. K., and A‐M. L.; visualization, E. M. K. S. M., E. M. A. and A‐M. L.; supervision, E. M. K., and A‐M. L.; project administration, S. M. and E. M. A.; funding acquisition, S. M., E. M. A., and A‐M. L.

## Conflict of Interests

The authors declare no conflict of interest.

## Supporting information

As a service to our authors and readers, this journal provides supporting information supplied by the authors. Such materials are peer reviewed and may be re‐organized for online delivery, but are not copy‐edited or typeset. Technical support issues arising from supporting information (other than missing files) should be addressed to the authors.

Supporting Information
