# Supplementary material for: Molecular Insights Into β‐Glucuronidase Inhibition by Alhagi Graecorum Flavonoids: A Computational and Experimental Approach
Source: ChemistryOpen. 2024 Nov 19;14(3):e202400325. doi: 10.1002/open.202400325 (PMC11891452; doi:10.1002/open.202400325)
Supplement: Supplementary file 1 — Supporting Information [file OPEN-14-e202400325-s001.pdf]

ChemistryOpen

Supporting Information

Molecular Insights Into β-Glucuronidase Inhibition by *Alhagi Graecorum* Flavonoids: A Computational and Experimental Approach

Emadeldin M. Kamel,* Saleh Maodaa, Esam M. Al-Shaebi, and Al Mokhtar Lamsabhi

Molecular Insights Into β-Glucuronidase Inhibition by *Alhagi Graecorum* Flavonoids: A Computational and Experimental Approach

Emadeldin M. Kamel [a]*, Saleh Maodaa [b], Esam M. Al-Shaebi [b], Al Mokhtar Lamsabhi [c,d]

[a] Chemistry Department, Faculty of Science, Beni-Suef University, Beni-Suef 62514, Egypt

[b] Department of Zoology, College of Science, King Saud University, PO Box -2455, Riyadh, 11451, Saudi Arabia

[c] Departamento de Química, Módulo 13, Universidad Autónoma de Madrid, Campus de Excelencia UAM-CSIC Cantoblanco, 28049 Madrid, Spain

[d] Institute for Advanced Research in Chemical Sciences (IAdChem), Universidad Autónoma de Madrid, 28049 Madrid, Spain

Contents

Cartesian coordinates of DFT-optimized isolated phenolics at the B3LYP level of theory…...2

DFT optimized geometries of isolated phenolics at the B3LYP level of theory………………8

Molecular Docking data…………………………………………………………………8

Spectral data of isolated flavonoids……………………………………………………....9

Figure S2. The ^{1}H-NMR spectrum of compound **1**………………………….………...…9

Figure S3. The ^{1}H-NMR spectrum of compound **1** (expanded)……………….……….....…9

Figure S4. The ^{13}C-NMR spectrum of compound **1**…………………………………………10

Figure S5. The ^{1}H-NMR spectrum of compound **2**………………………………………10

Figure S6. The ^{1}H-NMR spectrum of compound **2** (expanded)………………….……….....11

Figure S7. The ^{13}C-NMR spectrum of compound **2**…………………………………………11

Figure S8. The ^{1}H-NMR spectrum of compound **3**…………………………………………12

Figure S9. The ^{1}H-NMR spectrum of compound **3** (expanded)……………….……….....12

Figure S10. The ^{13}C-NMR spectrum of compound **3**………...……………………….....13

Figure S11. The ^{1}H-NMR spectrum of compound **4**……………………….…….……..13

Figure S12. The ^{13}C-NMR spectrum of compound **4**……………..……………...…….14

Figure S13. The ^{1}H-NMR spectrum of compound **5**………………………………..…….14

Figure S14. The ^{13}C-NMR spectrum of compound **5**……………………………………..15

Cartesian coordinates of DFT-optimized geometries of isolated flavonoids at the B3LYP level of theory

Chrysoeriol

C	-0.9101	0.5244	0.14885
C	-0.2825	1.7049	0.18015
C	-1.0453	2.9907	0.19565
C	-2.5346	2.7796	0.17225
C	-3.0655	1.4835	0.13995
O	-2.2749	0.3554	0.12805
C	-4.4459	1.2822	0.11825
C	-5.3087	2.3779	0.12865
C	-4.7906	3.6725	0.16075
C	-3.4091	3.8746	0.18255
O	-0.52303	4.0796	0.22387
C	-0.15208	-0.57689	0.13685
C	-0.55253	-1.69112	-0.60045
C	0.23826	-2.8401	-0.61286
C	1.42948	-2.87472	0.11193
C	1.82998	-1.76047	0.84914
C	1.03913	-0.61151	0.86163
O	-0.15076	-3.92261	-1.329
C	0.78153	-4.96242	-1.20566
O	2.19765	-3.99086	0.09983
O	-6.64971	2.18485	0.10758
O	-2.91201	5.13474	0.21379
H	0.81711	1.73042	0.19479
H	-4.85319	0.26069	0.09297
H	-5.47178	4.53617	0.16891
H	-1.49193	-1.66376	-1.17211
H	2.76945	-1.78779	1.42068

H	1.35491	0.26725	1.44306
H	0.44135	-5.839	-1.8012
H	1.7709	-4.61962	-1.58302
H	0.8724	-5.25398	-0.13538
H	2.97699	-3.82517	0.65658
H	-6.81503	1.22724	0.08664
H	-3.66171	5.75339	0.21632

Myricetin

C	0	-1.41387	0.04128	-0.02771
C	0	-0.78627	1.22178	0.00359
O	0	0.56825	1.25322	0.02162
C	0	-1.54907	2.50758	0.01909
O	0	-1.02679	3.59648	0.04731
C	0	-3.03837	2.29648	-0.00431
C	0	-3.91287	3.39148	0.00599
O	0	-3.41578	4.65162	0.03724
C	0	-5.29437	3.18938	-0.01581
C	0	-5.81247	1.89478	-0.04791
O	0	-7.15348	1.70173	-0.06898
C	0	-4.94967	0.79908	-0.05831
C	0	-3.56927	1.00038	-0.03661
O	0	-2.77867	-0.12772	-0.04851
C	0	-0.65585	-1.06001	-0.03971
C	0	-1.0563	-2.17424	-0.77701
C	0	-0.26551	-3.32321	-0.78941
O	0	-0.65453	-4.40572	-1.50556
C	0	0.92571	-3.35783	-0.06463
O	0	1.69388	-4.47398	-0.07672
C	0	1.32621	-2.24359	0.67259
O	0	2.48346	-2.27724	1.37661
C	0	0.53537	-1.09463	0.68507
H	0	0.89019	0.33619	0.00772
H	0	-4.16548	5.27027	0.03976

H	0	-5.97555	4.05305	-0.00765
H	0	-7.3188	0.74412	-0.08991
H	0	-5.35696	-0.22243	-0.08358
H	0	-1.99569	-2.14688	-1.34867
H	0	0.01763	-5.09856	-1.39172
H	0	2.47322	-4.30829	0.48002
H	0	2.59065	-1.4187	1.81955
H	0	0.85115	-0.21587	1.26651

Isorhamnetin

C	0	-1.2612	0.50082	0.14349
C	0	-0.6336	1.68132	0.17479
O	0	0.72092	1.71276	0.19282
C	0	-1.3964	2.96712	0.19029
O	0	-0.87413	4.05602	0.21851
C	0	-2.8857	2.75602	0.16689
C	0	-3.7602	3.85102	0.17719
O	0	-3.26311	5.11116	0.20843
C	0	-5.1417	3.64892	0.15539
C	0	-5.6598	2.35432	0.12329
O	0	-7.00081	2.16127	0.10222
C	0	-4.797	1.25862	0.11289
C	0	-3.4166	1.45992	0.13459
O	0	-2.626	0.33182	0.12269
C	0	-0.50318	-0.60047	0.13149
C	0	-0.90363	-1.7147	-0.60582
C	0	-0.11284	-2.86367	-0.61822
O	0	-0.50186	-3.94618	-1.33436
C	0	0.43043	-4.986	-1.21102
C	0	1.07838	-2.89829	0.10657
O	0	1.84655	-4.01444	0.09447
C	0	1.47888	-1.78405	0.84378
C	0	0.68804	-0.63509	0.85627
H	0	1.04286	0.79573	0.17891

H	0	-4.01281	5.72981	0.21096
H	0	-5.82288	4.51259	0.16355
H	0	-7.16613	1.20366	0.08128
H	0	-5.20429	0.23711	0.08761
H	0	-1.84302	-1.68734	-1.17748
H	0	0.09025	-5.86258	-1.80656
H	0	1.4198	-4.6432	-1.58838
H	0	0.52131	-5.27756	-0.14074
H	0	2.62589	-3.84875	0.65121
H	0	2.41835	-1.81137	1.41531
H	0	1.00382	0.24367	1.4377

Naringenin

C	0	-2.58729	0.31147	-0.72349
C	0	-1.64279	-0.87843	-0.71249
C	0	-2.45729	-2.15443	-0.86289
O	0	-2.17697	-3.06619	-1.60408
C	0	-3.65809	-2.12303	0.03801
C	0	-4.36109	-3.30443	0.31161
O	0	-3.95448	-4.47321	-0.24033
C	0	-5.47749	-3.28413	1.14921
C	0	-5.90169	-2.08433	1.71721
O	0	-6.98052	-2.06562	2.53685
C	0	-5.21409	-0.90293	1.44071
C	0	-4.10629	-0.91883	0.59331
O	0	-3.49249	0.28797	0.38361
C	0	-1.779	1.56987	-0.65939
C	0	-1.84808	2.49682	-1.69933
C	0	-1.09482	3.66929	-1.63962
C	0	-0.27265	3.91478	-0.53995
O	0	0.45905	5.05377	-0.48198
C	0	-0.20364	2.9879	0.50002
C	0	-0.95683	1.81536	0.44028
H	0	-3.18779	0.26496	-1.65944

H	0	-0.92408	-0.79244	-1.55796
H	0	-1.07688	-0.90245	0.2456
H	0	-3.17117	-4.2911	-0.78628
H	0	-6.02276	-4.21569	1.36107
H	0	-7.32041	-2.97357	2.60676
H	0	-5.54618	0.04369	1.89198
H	0	-2.49655	2.30324	-2.56651
H	0	-1.14919	4.40031	-2.45978
H	0	0.96224	5.04551	0.34959
H	0	0.44473	3.18155	1.36727
H	0	-0.90239	1.08431	1.26041

Tamarixetin

C	0	-1.41548	0.36473	-0.05746
C	0	-0.78788	1.54523	-0.02616
C	0	-1.55068	2.83103	-0.01066
C	0	-3.03998	2.61993	-0.03406
C	0	-3.57088	1.32383	-0.06636
O	0	-2.78028	0.19573	-0.07826
C	0	-4.95128	1.12253	-0.08806
C	0	-5.81408	2.21823	-0.07766
C	0	-5.29598	3.51283	-0.04556
C	0	-3.91448	3.71493	-0.02376
O	0	-1.0284	3.91993	0.01756
C	0	-0.65746	-0.73655	-0.06946
C	0	-1.05791	-1.85078	-0.80677
C	0	-0.26712	-2.99976	-0.81917
C	0	0.9241	-3.03438	-0.09438
C	0	1.3246	-1.92014	0.64283
C	0	0.53376	-0.77117	0.65532
O	0	1.69228	-4.15053	-0.10648
C	0	2.83802	-3.97493	0.68221
O	0	-0.65614	-4.08227	-1.53531
O	0	-7.15509	2.02519	-0.09873

O	0	-3.41739	4.97507	0.00748
O	0	0.56664	1.57667	-0.00813
H	0	-5.35857	0.10103	-0.11334
H	0	-5.97716	4.37651	-0.0374
H	0	-1.9973	-1.82342	-1.37842
H	0	2.26407	-1.94745	1.21437
H	0	0.84954	0.10759	1.23675
H	0	3.4522	-4.90278	0.657
H	0	3.43523	-3.1244	0.28381
H	0	2.53635	-3.7583	1.73142
H	0	0.01602	-4.77511	-1.42147
H	0	-7.32041	1.06758	-0.11967
H	0	-4.16709	5.59372	0.01001
H	0	0.88858	0.65964	-0.02204

DFT-optimized geometries of isolated flavonoids at the B3LYP level of theory

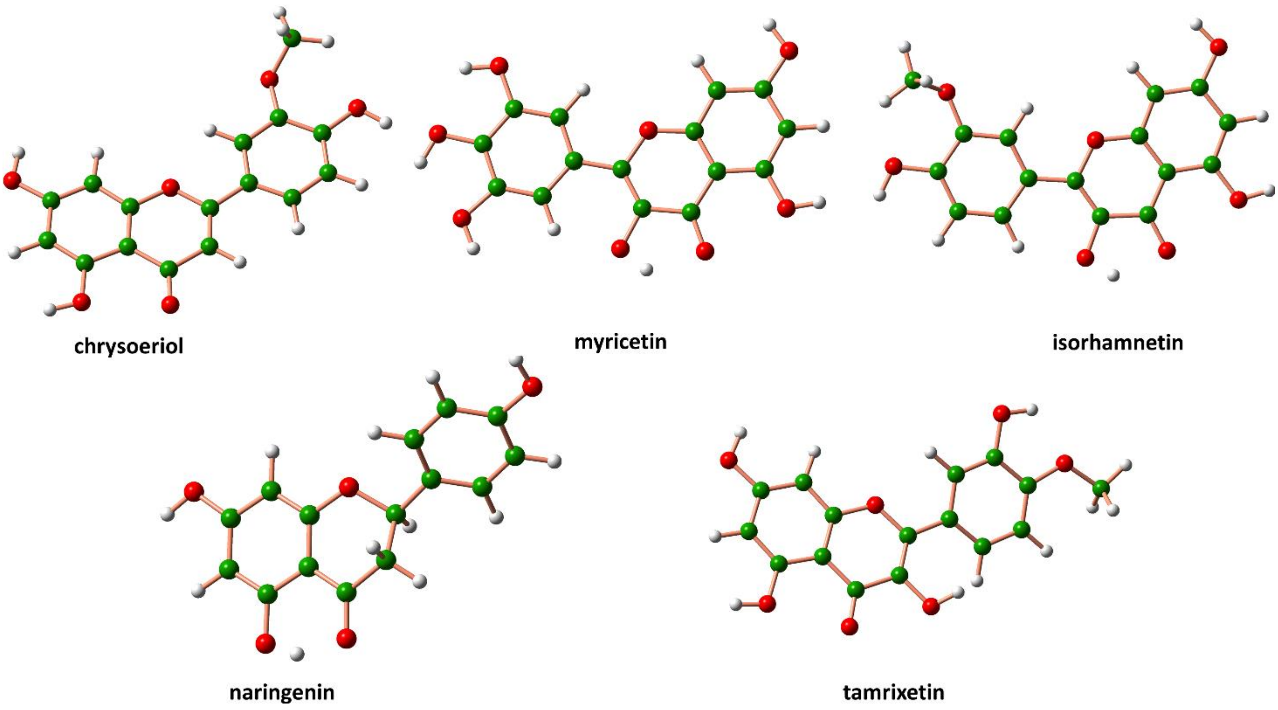

Figure S1. DFT optimized geometries of isolated phenolics at the B3LYP level of theory.

Molecular docking data

The grid box dimensions are center_x = -26.061, center_y = -23.252, and center_z = 1.935 and size_x = 34, size_y = 38, and size_z = 30.

Spectral data of isolated flavonoids

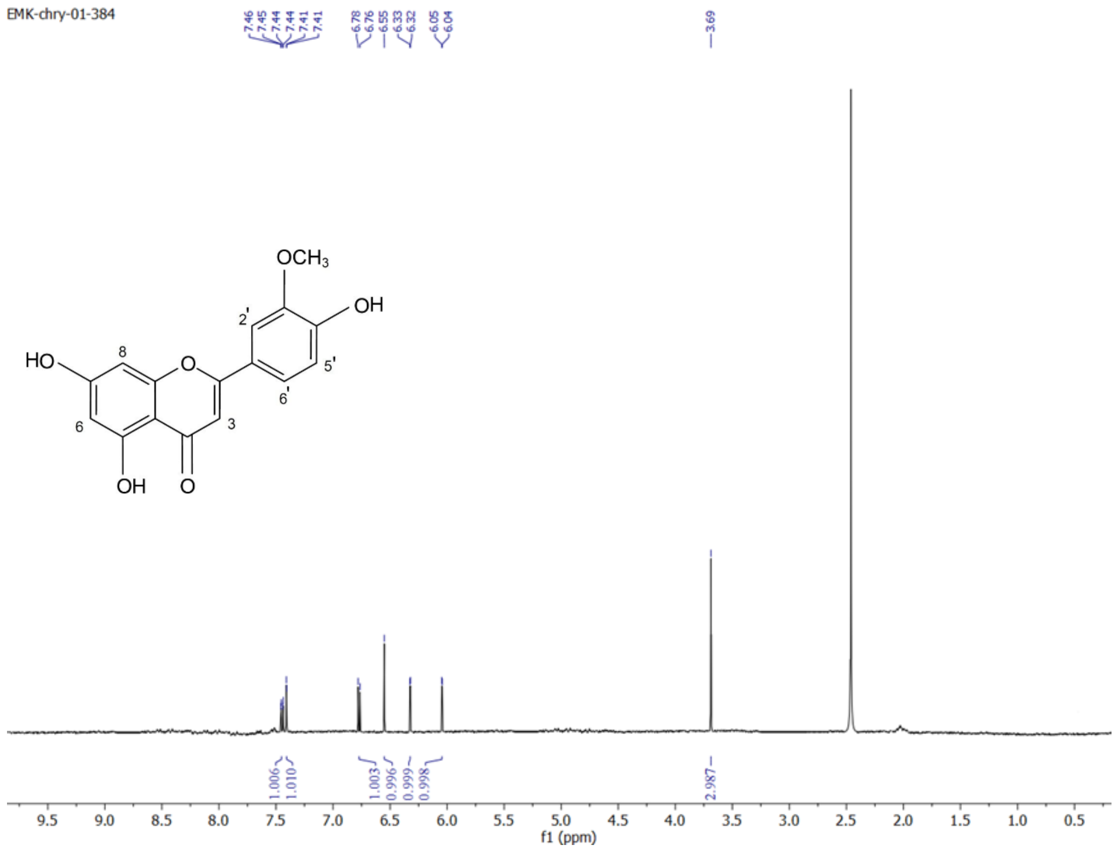

Figure S2. ^{1}H-NMR (DMSO-d_6) spectrum of compound **1**

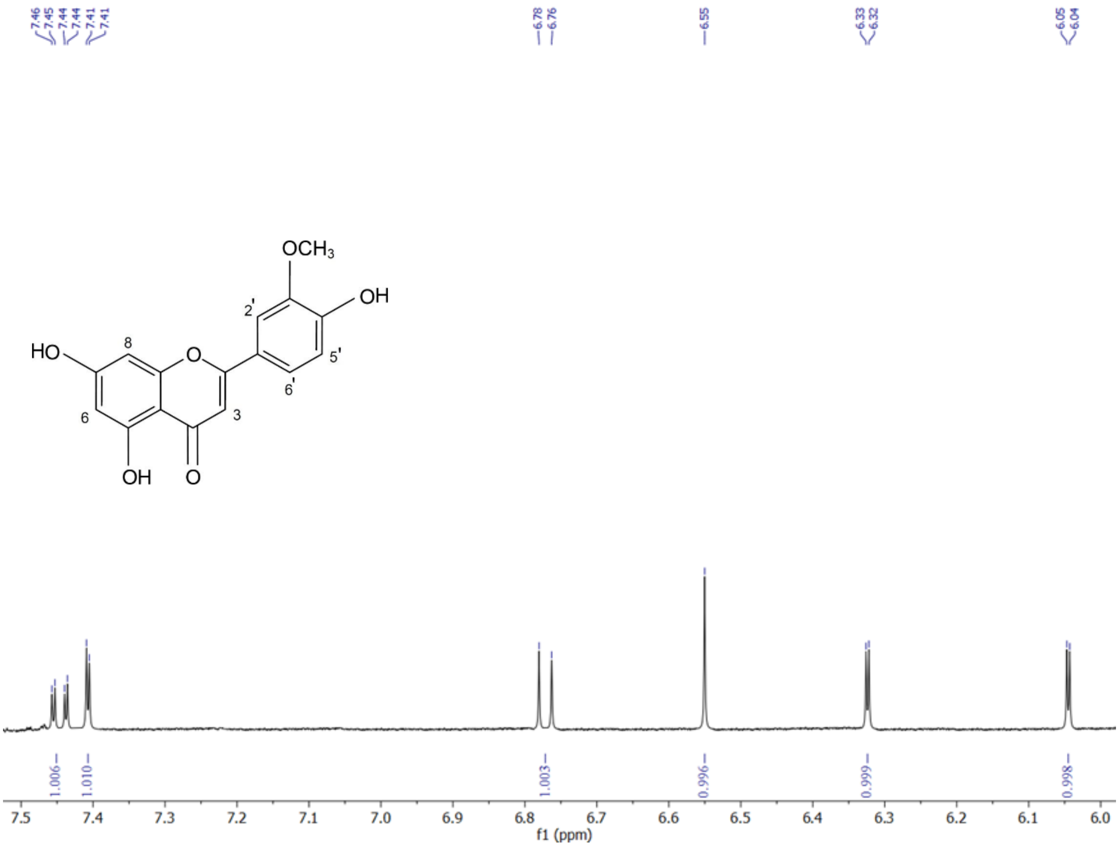

Figure S3. ^{1}H-NMR (DMSO-d_6) spectrum of compound **1** (expanded)

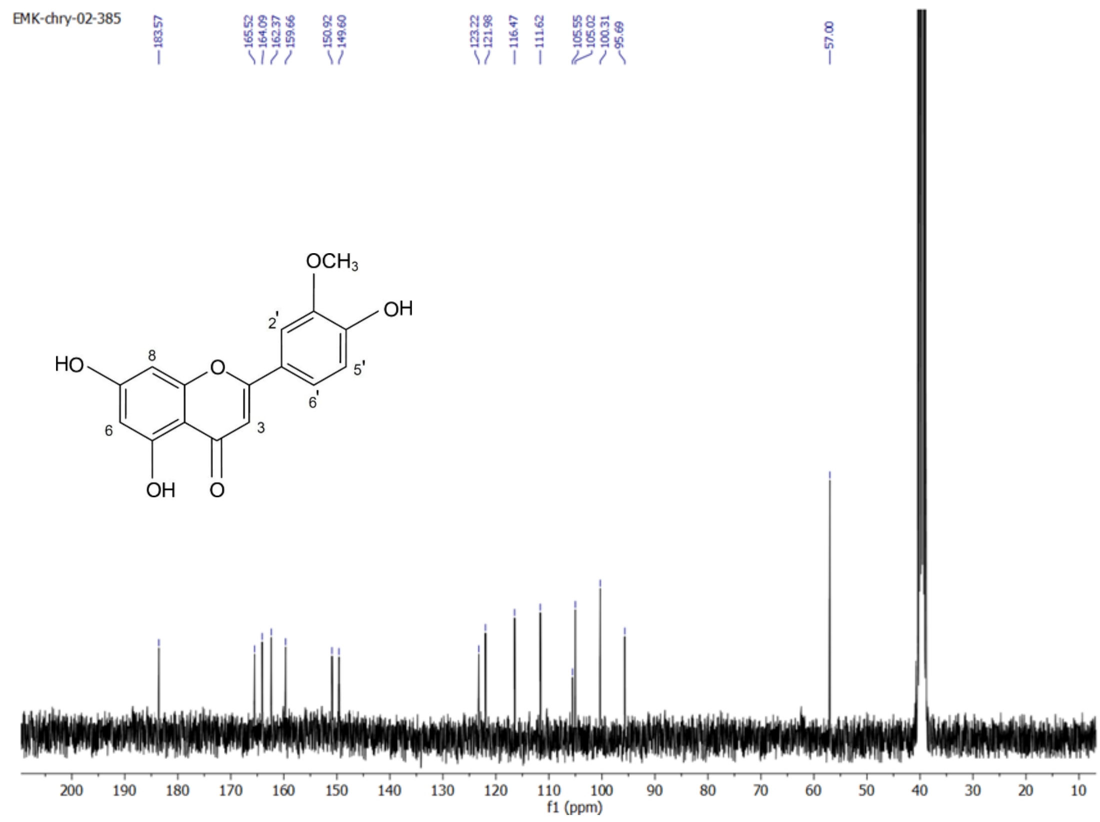

Figure S4. ^{13}C-NMR (DMSO-d$_6$) spectrum of compound **1**

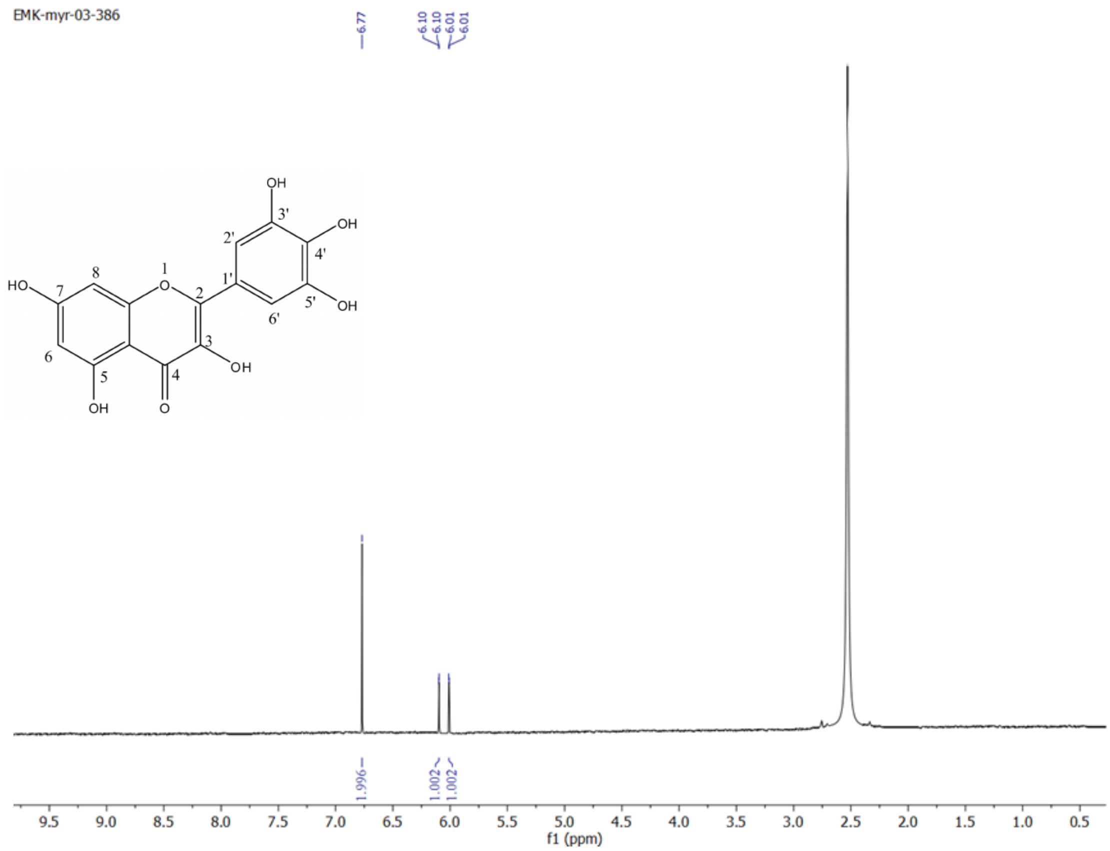

Figure S5. ^{1}H-NMR (DMSO-d$_6$) spectrum of compound **2**

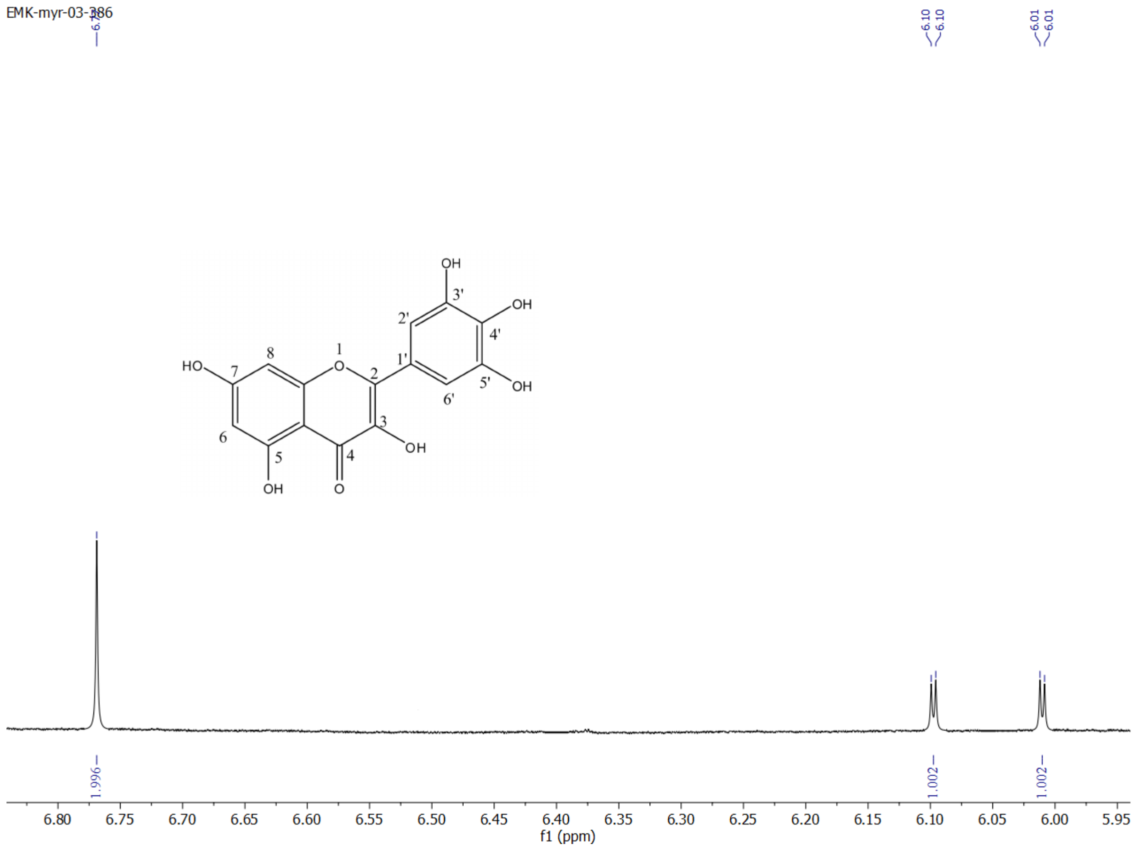

Figure S6. ^{1}H-NMR (DMSO-d$_6$) spectrum of compound **2** (Expanded)

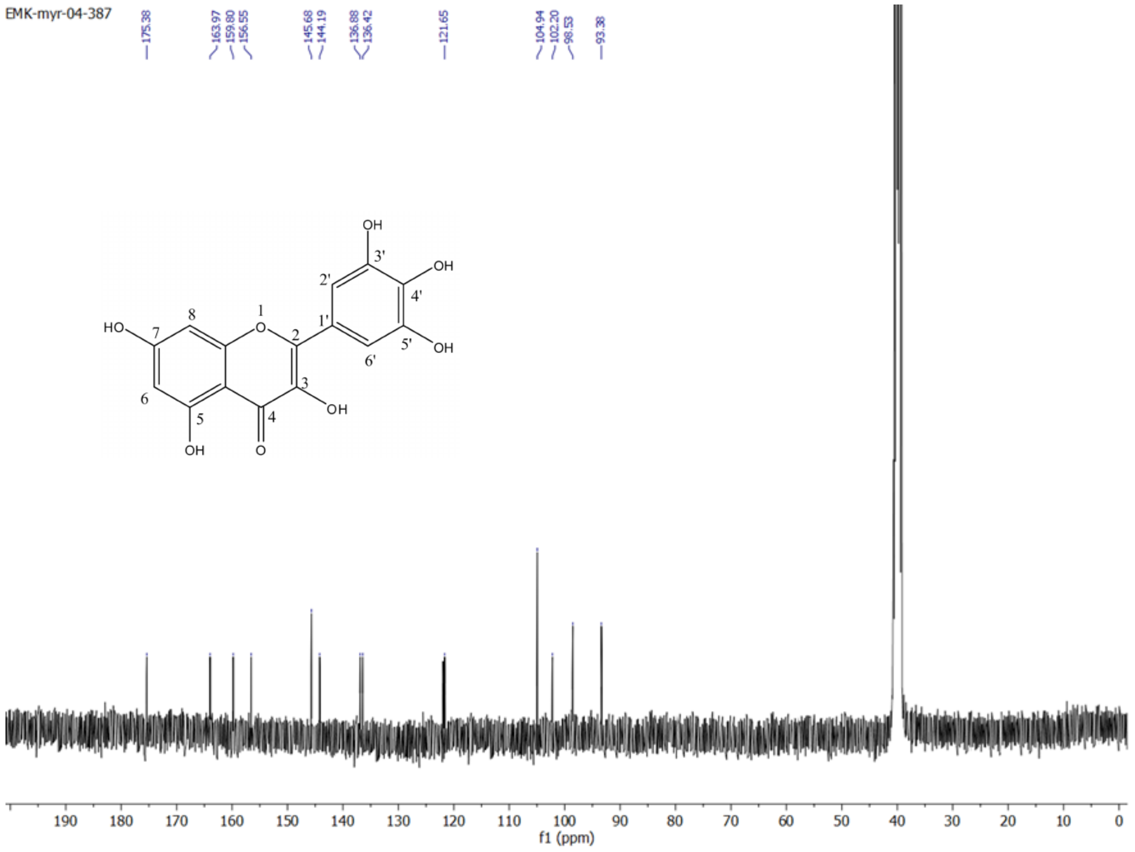

Figure S7. ^{13}C-NMR (DMSO-d$_6$) spectrum of compound **2**

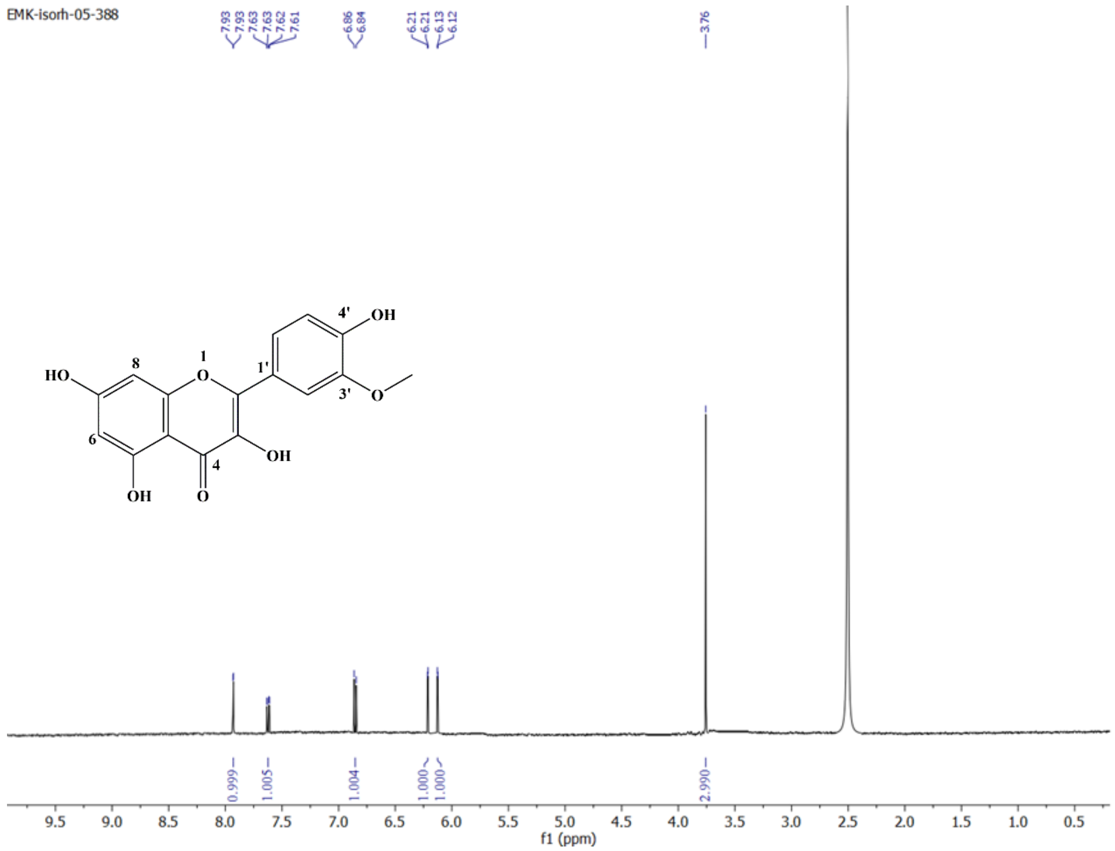

Figure S8. ^{1}H-NMR (DMSO-d$_6$) spectrum of compound **3**

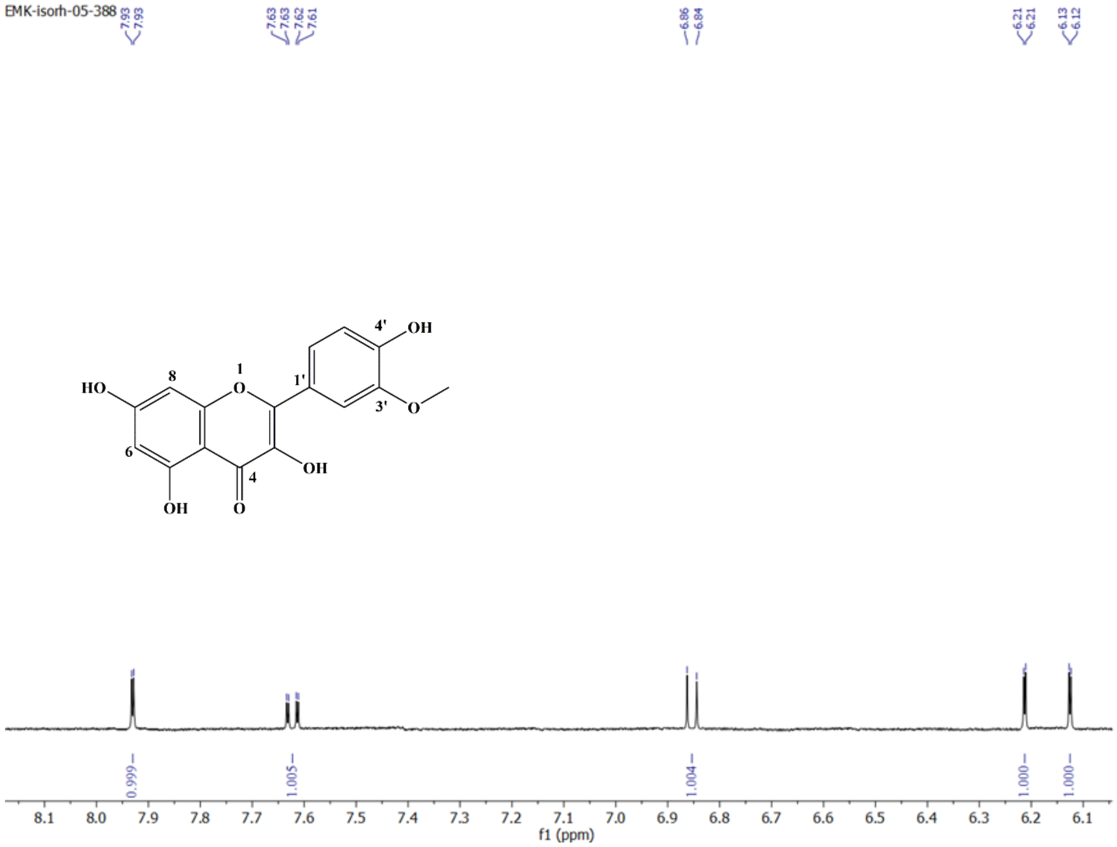

Figure S9. ^{1}H-NMR (DMSO-d$_6$) spectrum of compound **3** (expanded)

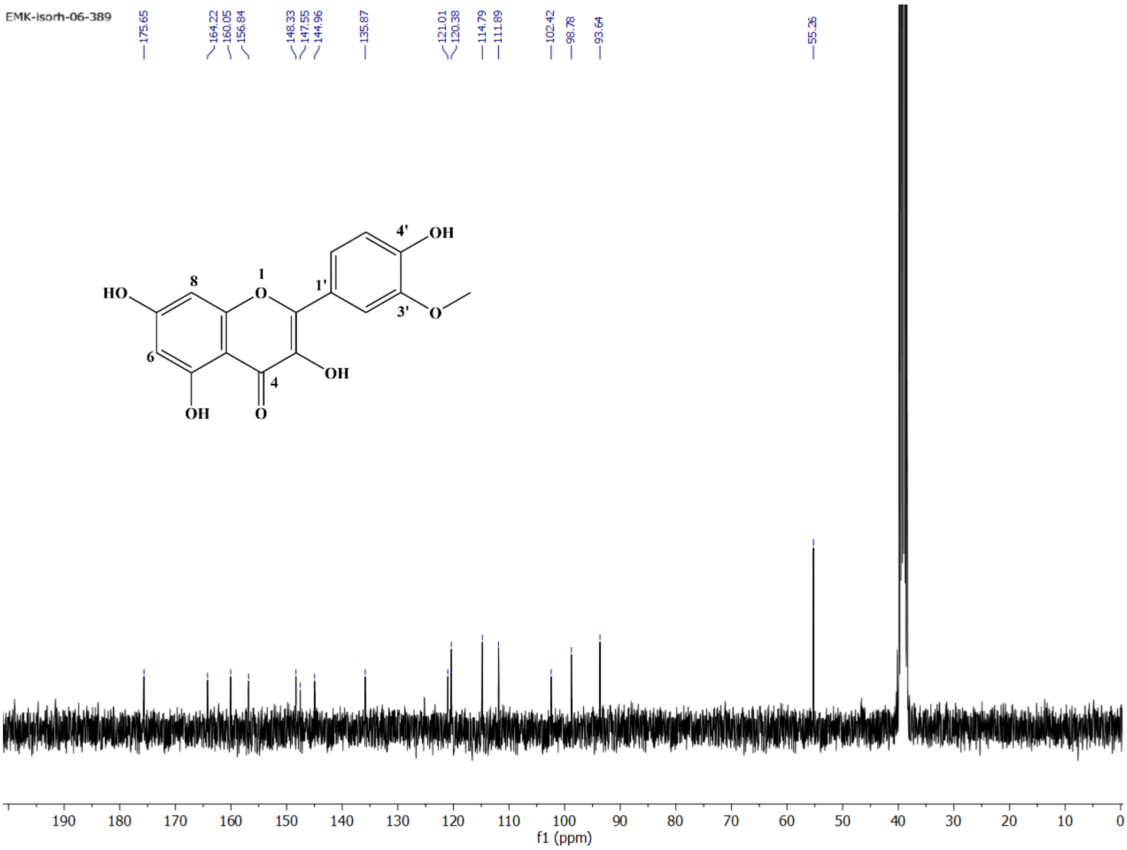

Figure S10. ^{13}C-NMR (DMSO-d_6) spectrum of compound **3**

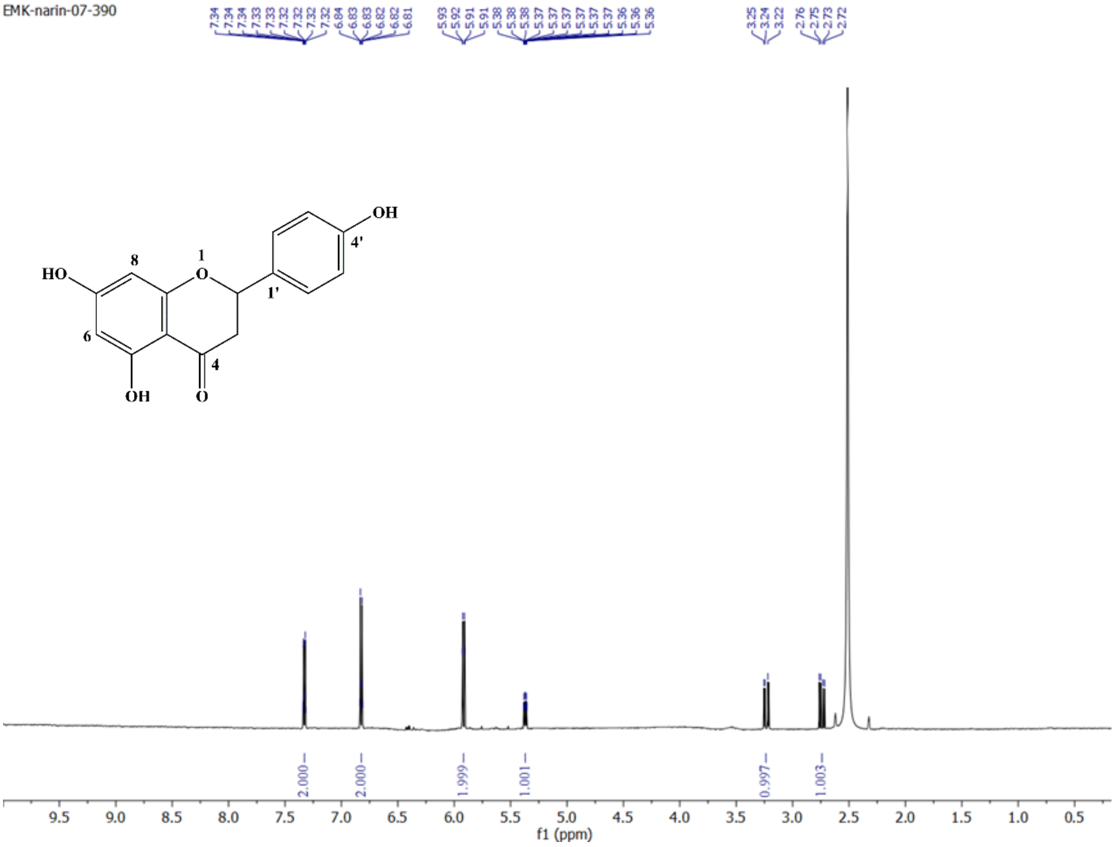

Figure S11. ^{1}H-NMR (DMSO-d_6) spectrum of compound **4**

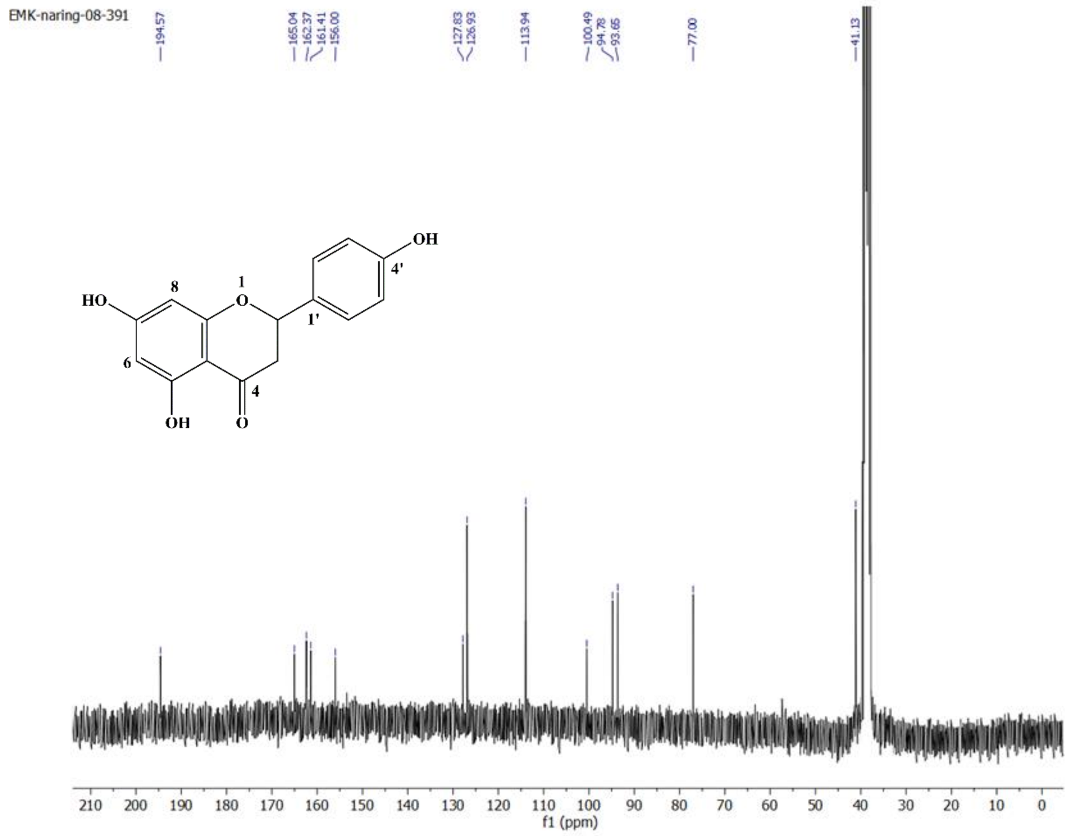

Figure S12. ^{13}C-NMR (DMSO-d_6) spectrum of compound **4**

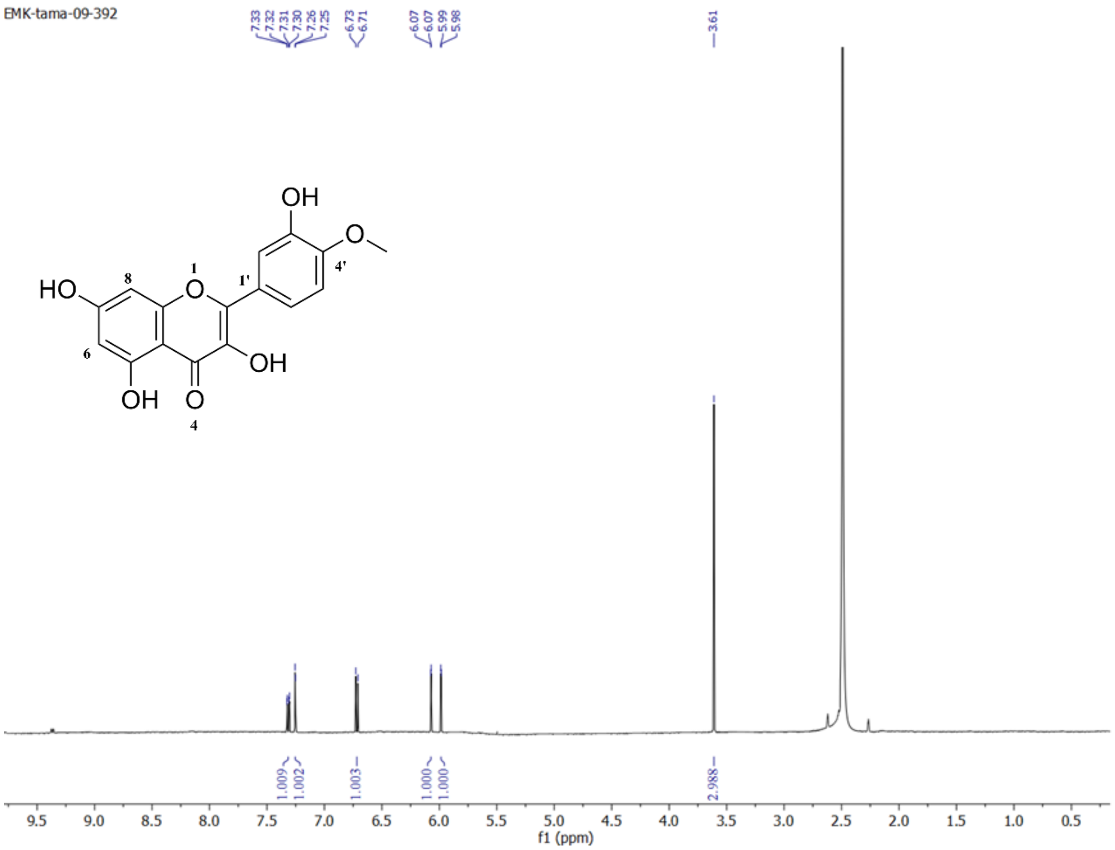

Figure S13. ^{1}H-NMR (DMSO-d_6) spectrum of compound **5**

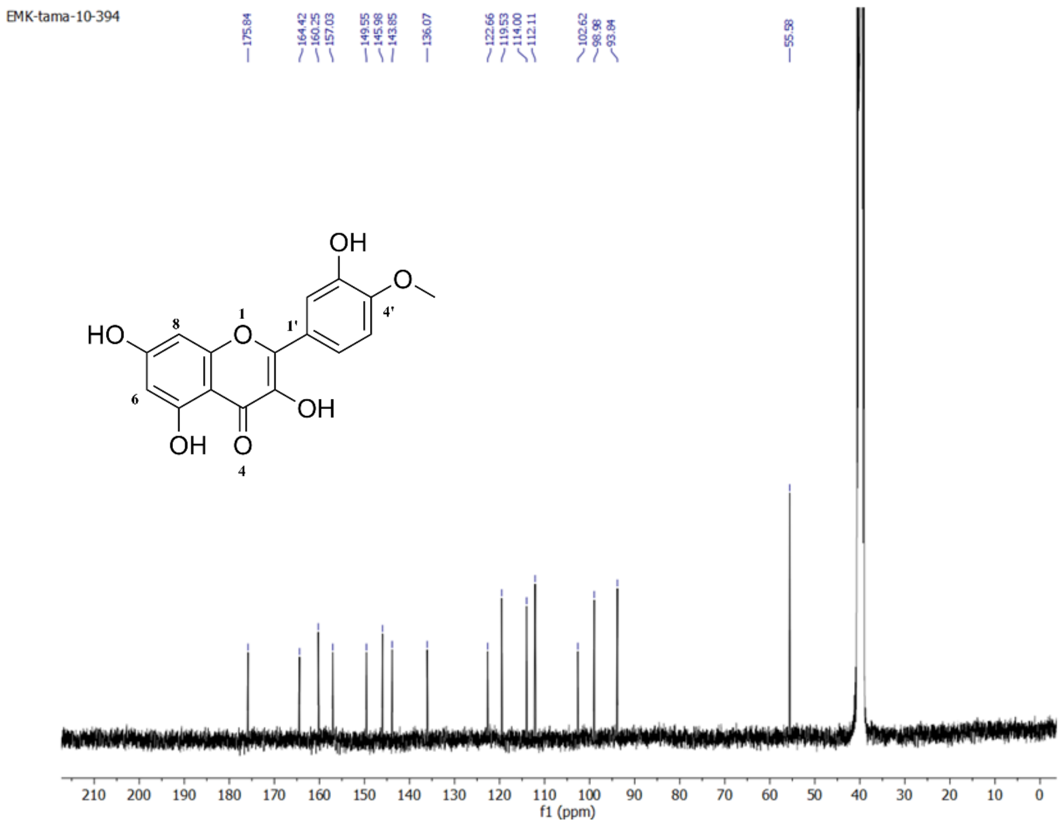

Figure S14. ^{13}C-NMR (DMSO-d$_6$) spectrum of compound **5**
